# *Mycoplasma fermentans* infection induces human necrotic neuronal cell death via IFITM3-mediated amyloid-β (1–42) deposition

**DOI:** 10.1038/s41598-023-34105-y

**Published:** 2023-04-26

**Authors:** Kyu-Young Sim, Yeongseon Byeon, So-Eun Bae, Taewoo Yang, Cho-Rong Lee, Sung-Gyoo Park

**Affiliations:** 1 Institute of Pharmaceutical Sciences, College of Pharmacy, Seoul National University, Seoul, Republic of Korea; 2 School of Life Sciences, Gwangju Institute of Science and Technology (GIST), Gwangju, Republic of Korea

**Keywords:** Cell biology, Immunology, Microbiology, Neuroscience

## Abstract

*Mycoplasma fermentans* is a proposed risk factor of several neurological diseases that has been detected in necrotic brain lesions of acquired immunodeficiency syndrome patients, implying brain invasiveness. However, the pathogenic roles of *M. fermentans* in neuronal cells have not been investigated. In this study, we found that *M. fermentans* can infect and replicate in human neuronal cells, inducing necrotic cell death. Necrotic neuronal cell death was accompanied by intracellular amyloid-β (1–42) deposition, and targeted depletion of amyloid precursor protein by a short hairpin RNA (shRNA) abolished necrotic neuronal cell death. Differential gene expression analysis by RNA sequencing (RNA-seq) showed that interferon-induced transmembrane protein 3 (*IFITM3*) was dramatically upregulated by *M. fermentans* infection, and knockdown of IFITM3 abolished both amyloid-β (1–42) deposition and necrotic cell death. A toll-like receptor 4 antagonist inhibited *M. fermentans* infection-mediated *IFITM3* upregulation. *M*. *fermentans* infection also induced necrotic neuronal cell death in the brain organoid. Thus, neuronal cell infection by *M*. *fermentans* directly induces necrotic cell death through IFITM3-mediated amyloid-β deposition. Our results suggest that *M*. *fermentans* is involved in neurological disease development and progression through necrotic neuronal cell death.

## Introduction

Mycoplasmas are the smallest and simplest self-replicating prokaryotes. They are found everywhere in nature and are recognized as pathogens and cofactors of several diseases^1,2^. They belong to the Mollicutes class, and their small size and absence of a cell wall distinguish them from other bacteria. More than 100 species have been identified, and they usually display strict host and tissue specificities. However, despite clinical evidence implicating mycoplasmas in various neurological diseases^4–7^, mycoplasma-host cell interactions and pathogenicity remain poorly understood^3^.

*Mycoplasma fermentans* has been detected in and isolated from numerous tissues, including blood of human immunodeficiency virus (HIV)-infected patients; hence, it is considered an invasive opportunistic pathogen under immunocompromised conditions^4^. However, clinical studies also suggest that *M. fermentans* is a putative risk factor of certain neurological diseases, including chronic fatigue syndrome (CFS)^5^, Gulf War syndrome (GWS)^5^, amyotrophic lateral sclerosis (ALS)^6^, and autism spectrum disorder (ASD)^7^. Many studies have reported central nervous system (CNS) invasion by mycoplasmas, suggesting that it could negatively affect neuronal cells in various animals^8^. *M. gallisepticum* and *M. synoviae* have been isolated from brains of birds, and *M. bovis* has been isolated from brain tissue of calves^8^. Also, *M. fermentans* can directly disseminate into the CNS since it has been found in brain necrotic lesions of AIDS patients^9,10^. In addition, in vivo experiments detected *M. fermentans* DNA in the brain after the bacterium was intraperitoneally injected into nonhuman primates^11^ and intratracheally injected into hamsters^12^. *M. fermentans* was also identified in necrotizing lesions in lymph nodes, spleen, liver, and brain in non-AIDS patients who died of an acute flu-like illness^13,14^. In a study on *M. pneumoniae*, bacteria were detected in and isolated from cerebrospinal fluid (CSF) samples of patients presenting various neurological symptoms^15^, and fatal acute disseminated encephalomyelitis patients showed brain invasion by *M. pneumonia*^16^. This evidence suggests that infection with mycoplasmas, including *M. fermentans*, can result in invasion of the CNS and damage to neuronal cells, promoting pathogenesis in various human neurological diseases.

Even though *M. fermentans* has been suggested as an etiological factor for several human neurological diseases, interactions between *M. fermentans* and neuronal cells in neurological diseases remain poorly understood. In this study, cellular and molecular approaches were used to understand the neuropathological changes in patients infected with *M. fermentans*. We provide evidence for necrotic neuronal cell death caused by *M. fermentans* in both human neuronal cells and brain organoids. Although we used a brain organoid system instead of a mouse infection model because *M. fermentans* did not induce toll-like receptor 4 (TLR4)-mediated interferon-induced transmembrane protein 3 (*IFITM3*) upregulation and necrotic cell death in mouse neuronal cells, our cerebral organoid model system clearly supports *M. fermentans* infection-mediated necrotic neuronal cell death.

## Results

### *M.fermentans* can infect and replicate in SH-SY5Y cells to induce necrotic cell death

**Figure 1:**
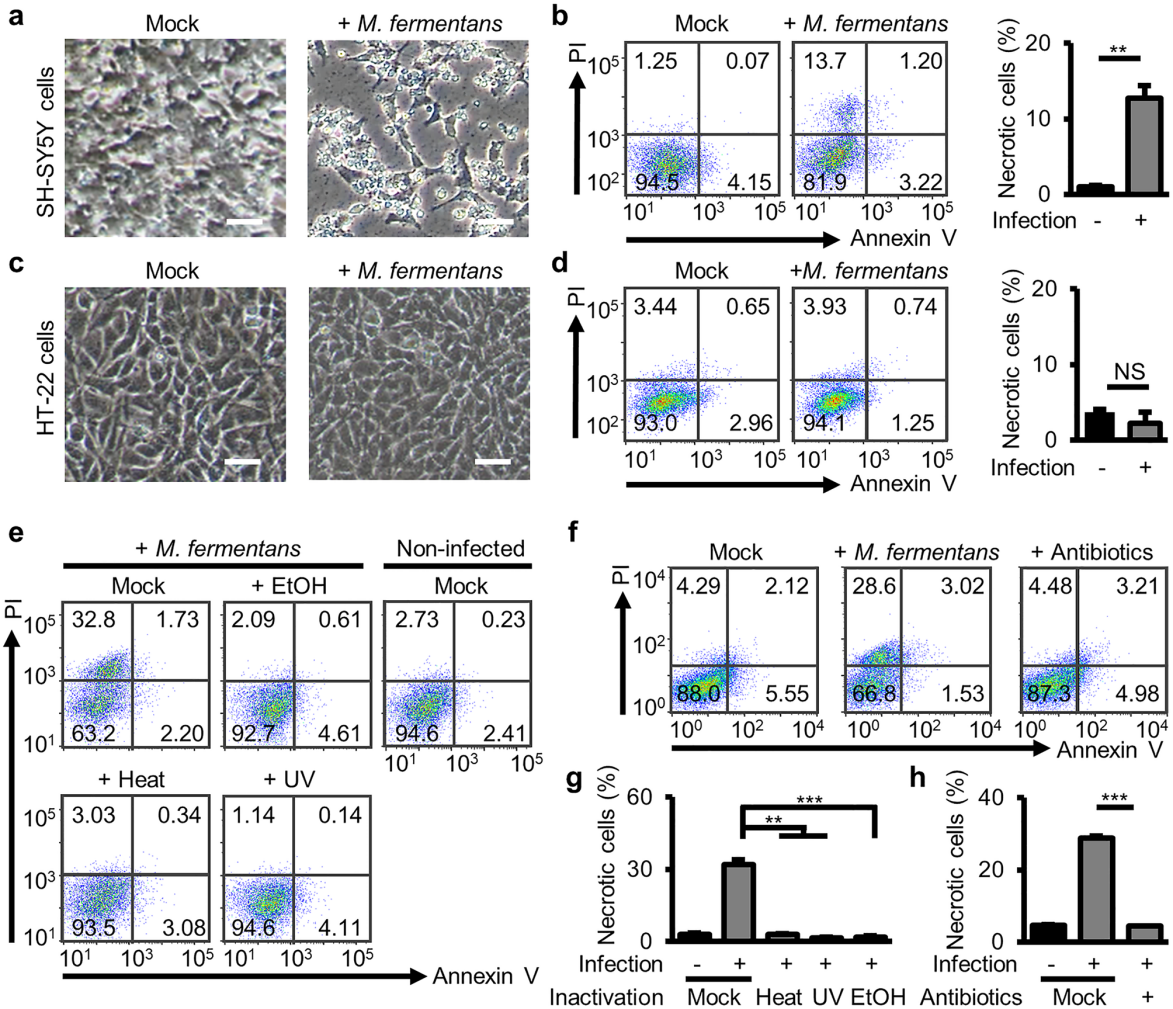
*M. fermentans* infection induces necrotic cell death in SH-SY5Y cells. (**a**) Representative microscopy images of mock- or *M. fermentans*-infected SH-SY5Y cells at 12 days postinfection (dpi). (**b**) Representative flow cytometry dot plots from Annexin V/propidium iodide (PI) apoptosis assays for SH-SY5Y cells from (**a**) and a bar graph of Annexin V-negative PI-positive cells (necrotic cells). (**c**) Representative microscopy images of HT-22 cells at 12 dpi. (**d**) Representative flow cytometry dot plots from Annexin V/PI apoptosis assays for HT-22 cells from (**c**) and a bar graph of Annexin V-negative PI-positive cells. (**e, f**) Representative flow cytometry dot plots from Annexin V/PI apoptosis assays for SH-SY5Y cells at 12 dpi after inactivation of *M. fermentans* with 70% EtOH, heating, UV irradiation (**e**), and mycoplasma antibiotics (**f**). (**g, h**) Bar graphs of Annexin V-negative PI-positive cells for data from (**e, f**). Bar graphs present mean values ± standard deviation (SD). Scale bars = 100 μm for images in (**a, c**); *p ≤ 0.05; **p ≤ 0.01; ***p ≤ 0.001; NS, not significant (unpaired Student’s t-test). Data are averages from three independent experiments.

To explore the pathogenic role of *M. fermentans* infection in neuronal cells, we infected human neuroblastoma SH-SY5Y cells and mouse hippocampal neuronal HT-22 cells with *M. fermentans*. To assess replication in neuronal cells, we used a MOI of 0.01. At 12 days post-infection (dpi), morphological changes, including significant cellular debris and a low cell count, were detected in infected SH-SY5Y cells (Fig. 1a). Although previous studies showed that *M. fermentans* infection promotes immortalization of Epstein-Barr virus (EBV)-infected human peripheral blood mononuclear cells^17^ and induces apoptosis in 32D cells^18^, infected SH-SY5Y cells showed more propidium iodide (PI)-staining and weaker annexin V-staining than uninfected controls (Fig. 1b), indicating that *M. fermentans* induces necrotic neuronal cell death, not apoptosis. However, HT-22 cells showed no morphological changes (Fig. 1c) or obvious cell death (Fig. 1d). To investigate infection and replication of *M. fermentans* in SH-SY5Y and HT-22 cells, intracellular and secreted *M. fermentans* DNA levels were measured by real-time quantitative PCR (qPCR). We detected intracellular and secreted *M. fermentans* DNA only in infected SH-SY5Y cells, not in HT-22 cells (Supplementary Fig. S1a, b). In addition, we infected another mouse neuroblastoma cell line (neuro2a) with *M. fermentans*. In contrast to HT-22 cells, we detected intracellular and secreted *M. fermentans* DNA in infected neuro2a cells (Supplementary Fig. S1c), but no necrotic cell death of mouse neuronal cells (Supplementary Fig. S1d). These results suggest that *M. fermentans* can directly infect and replicate in neuronal cell lines, but that necrotic cell death may be species-specific.

We also used phorbol 12-myristate 13-acetate (PMA)-induced differentiated SH-SY5Y cells to determine whether infection also affected differentiated neuronal cells (Supplementary Fig. S2a). Differentiated SH-SY5Y cells showed increased microtubule-associated protein 2 (*MAP2*) gene expression, extensive axon-like processes, and reduced cell growth compared with controls, consistent with previous studies (Supplementary Fig. S2b, c)^19^. At 19 dpi, *M. fermentans*-infected differentiated SH-SY5Y cells also exhibited necrotic cell death (Supplementary Fig. S2d–f). To determine the infectiousness of secreted *M. fermentans* derived from infected differentiated SH-SY5Y cells, we added conditioned medium from infected or noninfected differentiated SH-SY5Y cells into fresh differentiated SH-SY5Y cells at 19 dpi (Supplementary Fig. S2a). Conditioned medium derived from *M. fermentans*-infected differentiated SH-SY5Y cells induced necrotic cell death in fresh differentiated SH-SY5Y cells (Supplementary Fig. S2g, h). However, *M. fermentans* inactivated by heating, UV irradiation, and 70% ethanol treatment failed to induce necrotic cell death of SH-SY5Y cells (Fig. 1e–h). In addition, we performed in vitro drug testing by infecting with *M. fermentans* pretreated with mycoplasma antibiotics, and this drug treatment also failed to induce necrotic cell death. Overall, our results suggest that *M. fermentans* can infect and replicate in differentiated SH-SY5Y cells to induce necrotic cell death, and secreted *M. fermentans* then spreads into fresh cells to induce necrotic cell death. Indeed, clinical evidence suggests putative causal links between *M. fermentans* infection and various neurological diseases including CFS, GWS, ALS, and ASD (Supplementary Table S1)^5–7,20–24^. Therefore, our findings strengthen evidence indicating that *M. fermentans* may play a pathogenic role in human neurological diseases.

### Necrotic neuronal cell death caused by *M. fermentans* is mediated by intracellular Aβ_1−42_ deposition

**Figure 2:**
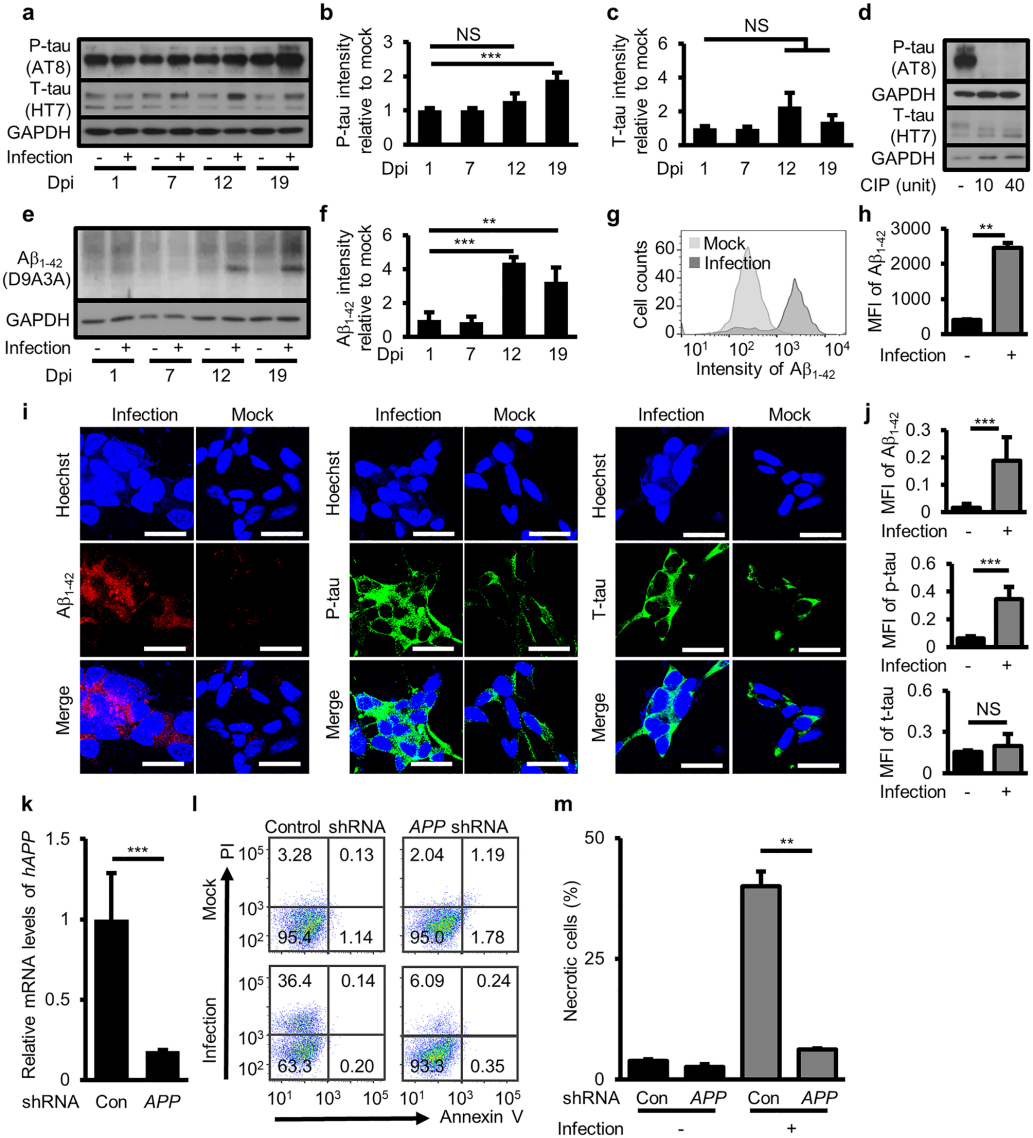
*M. fermentans*-mediated necrotic cell death induced by intracellular Aβ_1–42_ deposition. (**a**) Representative western blotting images from mock- or *M. fermentans*-infected differentiated SH-SY5Y cells using the indicated antibodies at 1, 7, 12, and 19 dpi. (**b, c**) Bar graphs of western blotting analysis data from (**a**) for the ratio of p-tau (**b**) and t-tau (**c**) to glyceraldehyde-3-phosphate dehydrogenase (GAPDH) relative to mock controls at each time point. (**d**) Representative western blotting images from *M. fermentans*-infected differentiated SH-SY5Y cells using the indicated antibodies at 19 dpi after calf intestinal phosphatase (CIP) treatment. (**e**) Representative western blotting images from mock- or *M. fermentans*-infected differentiated SH-SY5Y cells using the indicated antibodies at 1, 7, 12, and 19 dpi. (**f**) Bar graph of western blotting analysis data from (**e**) for the ratio of Aβ_1–42_ to GAPDH relative to mock controls at each time point. (**g**) Representative flow cytometry histogram of intracellular Aβ_1–42_ in mock- or *M. fermentans*-infected differentiated SH-SY5Y cells at 19 dpi. (**h**) Bar graph for data from (**g**). (**i**) Representative immunocytochemistry images from mock- or *M. fermentans*-infected differentiated SH-SY5Y cells using the indicated antibodies at 19 dpi. (**j**) Bar graph of mean fluorescence intensity (MFI) for each protein normalized by Hoechst intensity from (**i**). (**k**) Bar graph of h*APP* gene expression levels normalized against the *GAPDH* gene of h*APP* knockdown SH-SY5Y cells relative to control knockdown SH-SY5Y cells, determined by qPCR. (**l**) Representative flow cytometry dot plots from Annexin V/PI apoptosis assays for mock- or *M. fermentans*-infected indicated gene knockdown SH-SY5Y cells at 19 dpi. (**m**) Bar graph of Annexin V-negative PI-positive cells from (**l**). Original blots are presented in Supplementary Fig. S9. Con, control knockdown SH-SY5Y cells; *APP*, h*APP* knockdown SH-SY5Y cells. Bar graphs present mean values ± SD. Scale bars = 20 μm for (**i**); *p ≤ 0.05; **p ≤ 0.01; ***p ≤ 0.001; NS, not significant (unpaired Student’s t-test). Data are averages of three or more independent experiments.

Infectious neuro-pathogens can drive amyloidosis and thereby play a protective role in innate immunity in brain^25^. Therefore, we tested Aβ deposition caused by *M. fermentans*, and phosphorylated tau (p-tau) that is induced by Aβ deposition, in human neuronal cells^26^. To measure deposition of p-tau and Aβ_1−42_, we analyzed these proteins in differentiated SH-SY5Y cells at 7, 12, and 19 dpi using western blotting. The results showed that p-tau (Ser202, Thr205) was significantly increased at 19 dpi, but t-tau was not increased significantly (Fig. 2a– c). We also checked the quality of p-tau bands in the western blot; p-tau bands were confirmed by treating with calf intestinal phosphatase (CIP; Fig. 2d). In addition, intracellular Aβ_1−42_ levels were increased at 19 dpi (Fig. 2e, f). Increased intracellular Aβ_1−42_ was also confirmed using flow cytometry analysis (Fig. 2g, h). Immunocytochemical analysis also showed that both intracellular Aβ_1−42_ and p-tau were increased in differentiated SH-SY5Y cells at 19 dpi, but t-tau was not increased (Fig. 2i, j).

To validate necrotic cell death caused by Aβ_1–42_, we generated stable human amyloid precursor protein (h*APP*) knockdown and control knockdown SH-SY5Y cell lines using short hairpin RNA (shRNA) lentivirus, and we observed a decrease in h*APP* mRNA level in h*APP* knockdown SH-SY5Y cells compared with control cells (Fig. 2k). Necrotic neuronal cell death was dramatically inhibited in h*APP* knockdown cells at 19 dpi (Fig. 2l, m). These results indicate that *M. fermentans* induced intracellular Aβ_1–42_ deposition, and this caused necrotic cell death.

### *M. fermentans* infection induces Aβ_1–42_ accumulation and necrotic neuronal cell death in brain organoids

**Figure 3:**
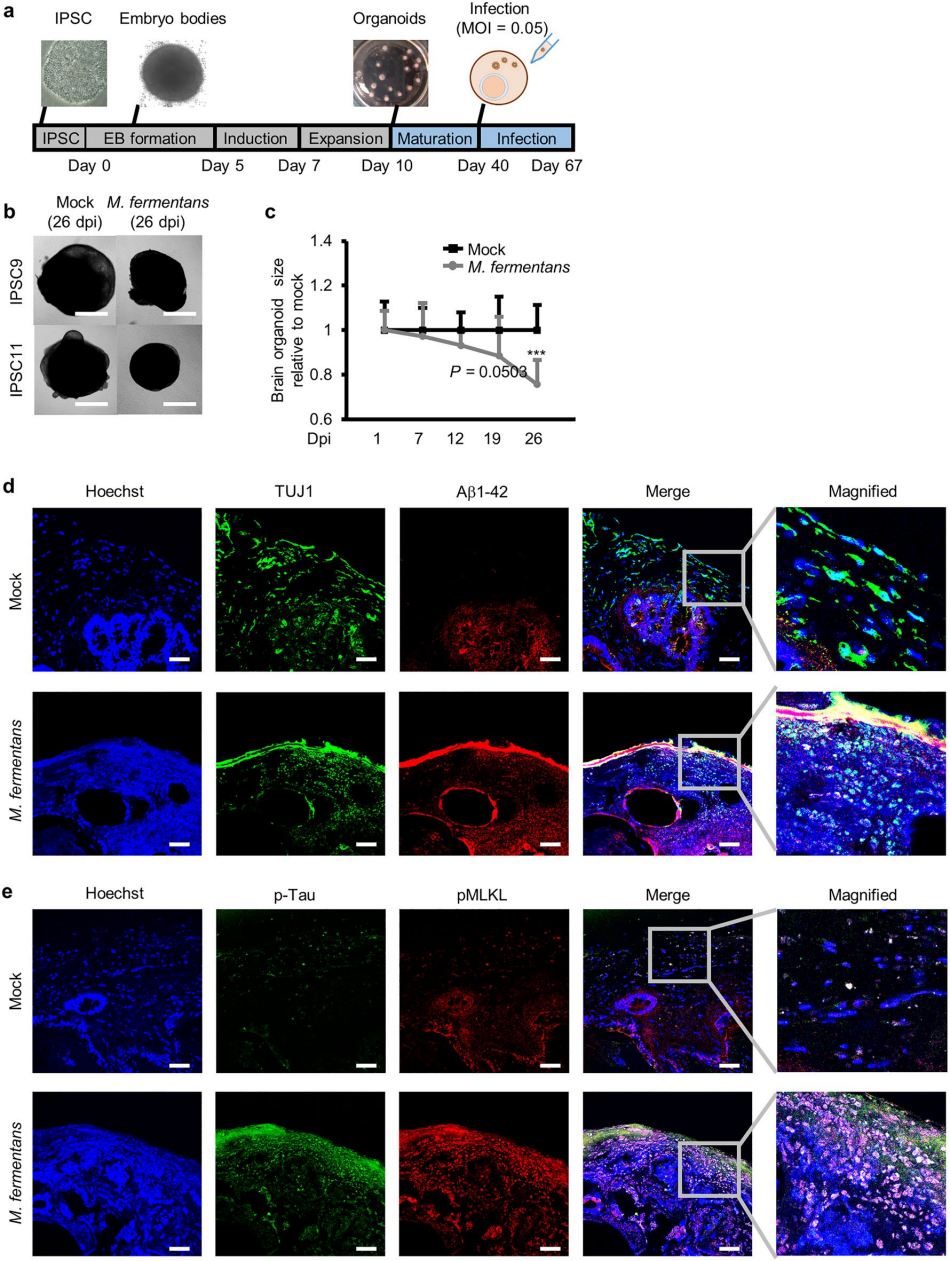
*M. fermentans* induces necrotic cell death accompanied by accumulation of Aβ_1–42_ and p-tau in human induced pluripotent stem cell (hIPSC)-derived brain organoids. (**a**) Schematic diagram of *M. fermentans* infection of maturated brain organoids derived from hIPSCs. (**b**) Representative microscopy images of mock- or *M. fermentans*-infected brain organoids at 26 dpi. (**c**) Dot plot of *M. fermentans*-infected brain organoid sizes relative to mock controls at each time point. (**d, e**) Representative immunohistochemistry images of mock- or *M. fermentans*-infected brain organoids using the indicated antibodies at 26 dpi. Scale bars = 1000 μm for (**b**) and 50 μm for (**d, e**). Results are expressed as mean ± SD; ***p ≤ 0.001 (unpaired Student’s t-test). Results are averaged from more than three independent experiments.

To evaluate the potential pathogenic impact of *M. fermentans* in a more physiologically relevant system, we utilized human brain organoids instead of a mouse infection model, since previous results showed that mouse neuronal cells are not susceptible to *M. fermentans*-induced necrotic cell death. We added *M. fermentans* to human brain organoids at 40 days (Fig. 3a). At 26 dpi, infected brain organoids were decreased in size compared with controls (Fig. 3b, c). To confirm the infection and replication of *M. fermentans* in brain organoids, intracellular and secreted *M. fermentans* DNA levels were measured by qPCR. We detected intracellular and secreted *M. fermentans* DNA only in *M. fermentans*-infected brain organoids at 26 dpi (Supplementary Fig. S3a–d). We conducted immunohistochemical analysis to confirm necrotic cell death and Aβ_1–42_ deposition in brain organoids at 26 dpi. After 67 days brain organoids exhibited a ventricular-like structure containing packed SOX2-positive neuronal progenitors, with a beta-tubulin III (TUJ1)-positive neuronal layer at the outer border (Supplementary Fig. S3e)^27^. Additionally, the infected brain organoids contained more Aβ_1–42_ in the disrupted TUJ1 and region around the outer border of brain organoids (Fig. 3d), indicating that *M. fermentans* can induce necrotic cell death through Aβ_1–42_ deposition in mature neurons of brain organoids. We also confirmed increased p-tau and phosphorylated mixed lineage kinase domain-like (pMLKL) protein in infected brain organoids (Fig. 3e). Collectively, these results suggest that *M. fermentans* induces necrotic neuronal cell death by inducing Aβ_1–42_ deposition in brain organoids as well as human neuronal cell lines. Our results also suggest that amyloid pathology can also induce hyperphosphorylated tau as well as necrotic neuronal cell death through pMLKL^26^.

### Differential gene expression induced by *M. fermentans* infection

**Figure 4:**
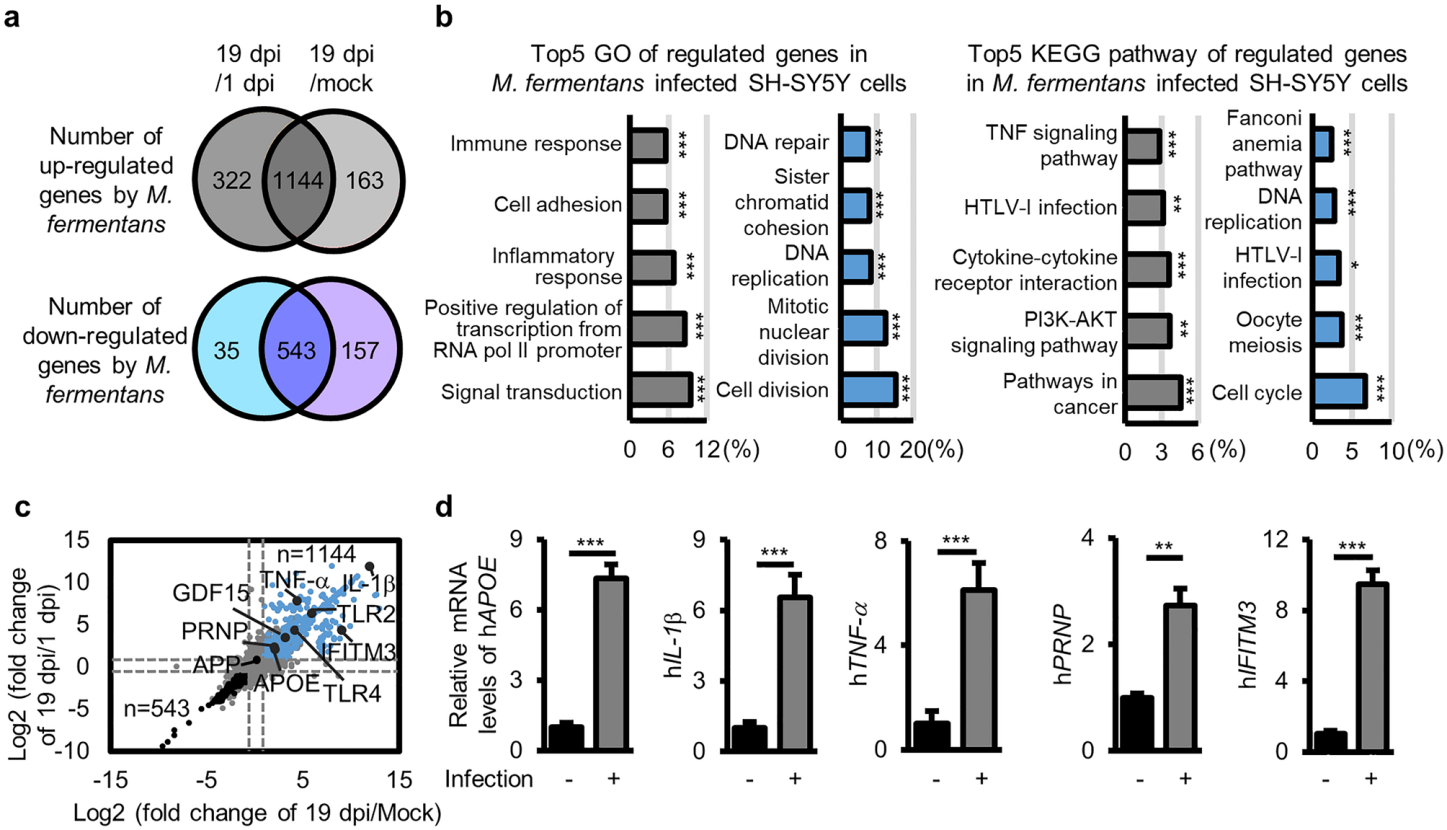
Differential gene expression induced by *M. fermentans* infection. (**a**) Venn diagram of the number of upregulated (grey, fold change ≥ 2) or downregulated (blue, fold change ≤ 2) genes of *M. fermentans*-infected differentiated SH-SY5Y cells at 19 dpi compared to 1 dpi or mock controls, determined by RNA-seq. (**b**) Gene Ontology (GO) biological process and Kyoto Encyclopedia of Genes and Genomes (KEGG) pathway categories related to upregulated (left grey panel) and downregulated (right blue panel) genes. All GO and KEGG groups were identified according to the EASE score (*p* ≤ 0.05). (**c**) Scatter plots of RNA-seq analysis data from (**a**). (**d**) Bar graph of indicated gene expression levels normalized against the *GAPDH* gene in *M. fermentans*-infected SH-SY5Y cells relative to mock-infected SH-SY5Y cells at 19 dpi, determined by qPCR. Bar graphs present mean values ± SD; *p ≤ 0.05; **p ≤ 0.01; ***p ≤ 0.001 (unpaired Student’s t-test). Data are averages of three or more independent experiments.

We sought to identify the pathways involved in intracellular Aβ_1–42_ deposition and necrotic cell death in *M. fermentans*-infected differentiated SH-SY5Y cells using RNA sequencing (RNA-seq) analysis. To identify genes implicated in necrotic cell death resulting from *M. fermentans* infection, we compared gene expression profiles between *M. fermentans*-infected samples collected at 19 dpi, when necrotic cell death was observed, and mock and 1 dpi samples, which did not display necrotic cell death. Analysis of 1dpi samples was conducted to distinguish genes associated with *M. fermentans* infection from those not related to necrotic cell death. Therefore, overlapping regions in the Venn diagram indicate genes that are specifically upregulated or downregulated in response to *M. fermentans* infection at 19 dpi and associated with necrotic cell death (Fig. 4a). We identified 1,144 upregulated genes and 543 downregulated genes after induction of necrotic cell death by *M. fermentans* infection (Fig. 4a), as well as the top five biological process categories and Kyoto Encyclopedia of Genes and Genomes (KEGG) pathways associated with either upregulated or downregulated genes (Fig. 4b). The top enriched pathways linked to downregulated genes included cell cycle, cell division, and DNA replication (Fig. 4b), while the top enriched pathways associated with upregulated genes included signal transduction, inflammatory response, immune response, cytokine-cytokine receptor interaction, and tumor necrosis factor (TNF) signaling (Fig. 4b). Notably, inflammatory responses and TNF signaling are central pathways of cognitive decline and Aβ_1–42_ deposition^28^. Interestingly, we found that growth differentiation factor 15 (GDF15), a blood marker for myalgic encephalomyelitis (ME)/CFS^29^ and mitochondria dysfunction^30^, was upregulated in *M. fermentans*-infected cells (Fig. 4c). We also identified upregulated genes known to be associated with AD pathogenesis, namely *TNF-α*, apolipoprotein E (*APOE*), prion protein (*PRNP*), *IFITM*3, and interleukin-1β (*IL-1β*) (Fig. 4c). These upregulated genes were confirmed by measuring mRNA levels in *M. fermentans*-infected SH-SY5Y cells by qPCR at 19 dpi (Fig. 4d). TNF and IL-1β are key proinflammatory cytokines in neuroinflammation and degenerative conditions^28,31^, and APOE interacts with Aβ and promotes the aggregation and pathology of Aβ^32^. PRNP encodes PrP, a receptor for internalization of oligomeric Aβ, to induce tau hyperphosphorylation and necrotic cell death^26,33^. IFITM3 in neurons and astrocytes binds to γ-secretase and upregulates its activity, thereby increasing the production of Aβ_1–42_^34^. IFITM3 is known to play a role in restricting infection by some viruses and bacteria, such as SARS-CoV-2 and *Mycobacterium tuberculosis*^35,36^. To investigate the potential positioning of genes (Fig. 4d) within the signaling pathway involved in *M. fermentans*-induced Aβ_1–42_ deposition, we performed experiments to assess the effect of h*APP* knockdown on their expression. The results revealed that knockdown of h*APP* inhibited h*IL-1β* and h*PRNP* expression in *M. fermentans*-infected SH-SY5Y cells, while h*APOE* expression was upregulated (Supplementary Fig. S4). These findings provide further insights into the potential roles of these genes in the signaling pathway, and suggest their potential as upstream or downstream components.

### *M. fermentans* induces IFITM3-mediated Aβ_1−42_ accumulation, resulting in necrotic neuronal cell death

**Figure 5:**
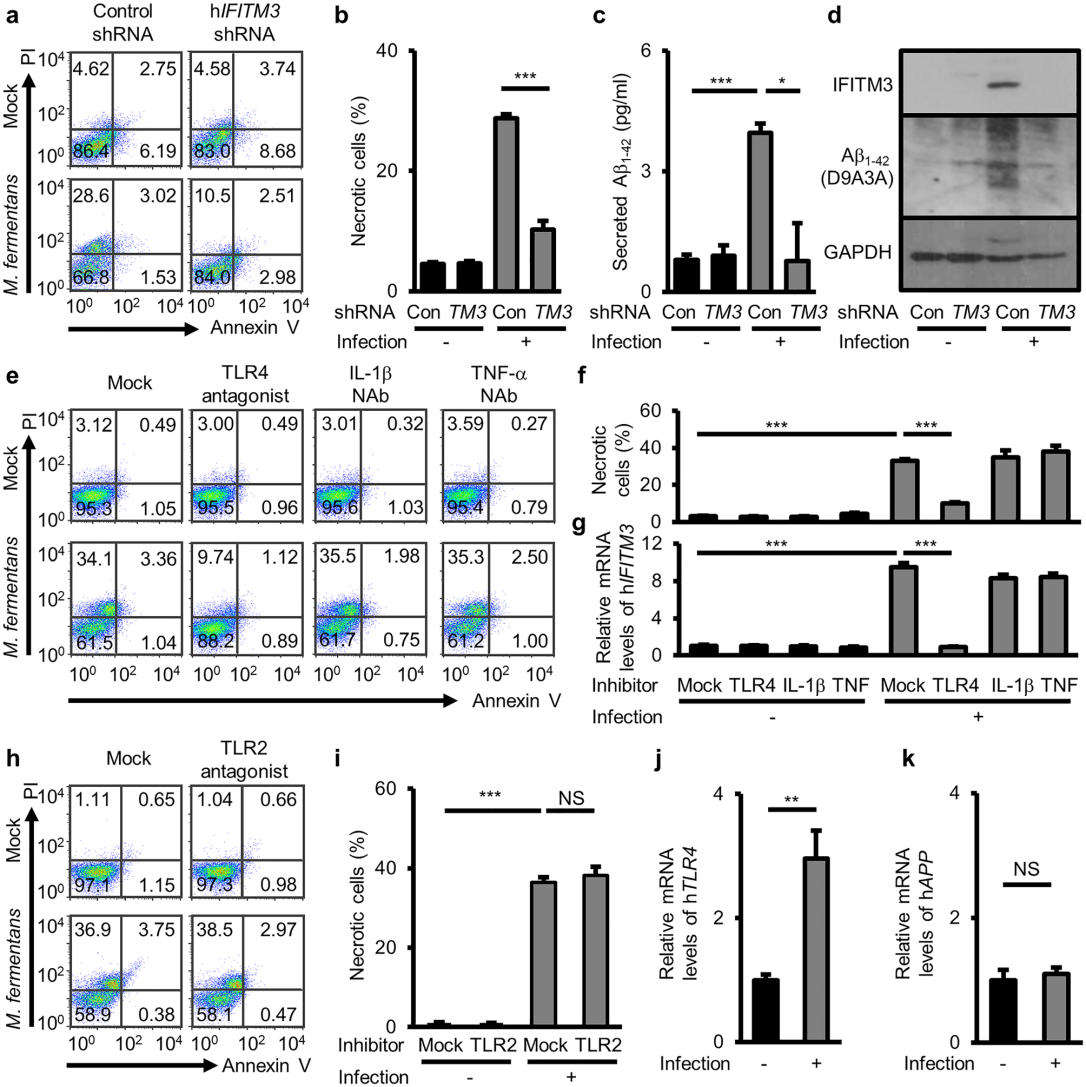
*M. fermentans* induces IFITM3-mediated Aβ_1-42_ accumulation, resulting in necrotic neuronal cell death. (**a**) Representative flow cytometry dot plots from Annexin V/PI apoptosis assays for mock- or *M. fermentans*-infected indicated gene knockdown SH-SY5Y cells at 12 dpi. (**b**) Bar graph of Annexin V-negative PI-positive cells for data from (**a**). (**c**) Quantification of secreted Aβ_1−42_ in the SH-SY5Y culture medium from (**a**) determined by ELISA. (**d**) Representative western blotting image from mock- or *M. fermentans*-infected indicated gene knockdown SH-SY5Y cells using the indicated antibodies at 12 dpi. (**e**) Representative flow cytometry dot plots from Annexin V/PI apoptosis assays of mock- or *M. fermentans-*infected SH-SY5Y cells after TLR4 antagonist or the indicated neutralizing antibody (NAb) treatment. (**f**) Bar graph of Annexin V-negative PI-positive cells from (**e**). (**g**) Bar graph of h*IFITM3* gene expression levels normalized against the *GAPDH* gene in infected SH-SY5Y cells from (**e**). (**h**) Representative flow cytometry dot plots from Annexin V/PI apoptosis assays of mock- or *M. fermentans*-infected SH-SY5Y cells after treatment with TLR2 antagonist. (**i**) Bar graph of Annexin V-negative PI-positive cells from (**h**). (**j, k**) Bar graphs of gene expression levels of h*TLR4* (**j**) and h*APP* (**k**) in infected SH-SY5Y cells at 12 dpi, which were determined by qPCR and normalized against the *GAPDH* gene. Original blots are presented in Supplementary Fig. S9. Con, control knockdown SH-SY5Y cells; *TM3*, h*IFITM3* knockdown SH-SY5Y cells; NAb, neutralizing antibody. Bar graphs present mean values ± SD; *p ≤ 0.05; **p ≤ 0.01; ***p ≤ 0.001 (unpaired Student’s t-test). Data are averages of three or more independent experiments.

To confirm the function of IFITM3 in Aβ_1–42_ deposition, we generated stable *IFITM3* knockdown SH-SY5Y cells using shRNA lentivirus and infected these with *M. fermentans*. To evaluate the impact of IFITM3 on mycoplasma infection, we conducted experiments to measure *M. fermentans* growth in cells with *IFITM3* knockdown. The results revealed a significantly higher infection level in cells with *IFITM3* knockdown than in control knockdown cells (Supplementary Fig. S5). Moreover, at 12 dpi, *M. fermentans*-infected *IFITM3* knockdown cells showed lower necrotic cell death than the control cells, although *M. fermentans* levels were higher in *IFITM3* knockdown cells (Fig. 5a, b). ELISA demonstrated that *M. fermentans* increased Aβ_1–42_ secretion, and that this was reduced by *IFITM3* knockdown (Fig. 5c). Furthermore, using western blotting, we confirmed that IFITM3 and intracellular Aβ_1–42_ protein levels were increased by *M. fermentans* infection, and that the levels of intracellular Aβ_1–42_ proteins were reduced in *IFITM3* knockdown cells (Fig. 5d).

A previous study showed that TLR2 and TLR4 ligands, and *Mycobacterium tuberculosis* infection, trigger IFITM3 to restrict mycobacterial growth^35^. RNA-seq data showed that *TLR2* and *TLR4* gene expression levels were increased in SH-SY5Y cells by *M. fermentans* at 19 dpi (Fig. 4c). Because proinflammatory cytokines can be associated with IFITM3 expression^35^, we explored the roles of IL-1β and TNF-α in IFITM3 upregulation during *M. fermentans* infection. However, neutralization of these cytokines did not affect *IFITM3* upregulation, while the TLR4 antagonist inhibited necrotic cell death (Fig. 5e, f) and *IFITM3* upregulation (Fig. 5g). A TLR2 antagonist had no inhibitory effect on necrotic neuronal cell death caused by *M. fermentans* infection (Fig. 5h, i). Therefore, we further investigated the effect of *M. fermentans* infection and found a significant upregulation of h*TLR4* mRNA levels (Fig. 5j). Interestingly, we did not observe any significant change in the h*APP* mRNA expression level, as confirmed by both RNA-seq (Fig. 4c) and qPCR (Fig. 5k). These results suggest that the observed increase in Aβ_1–42_ production was not due to substrate upregulation, but rather to increased γ-secretase activity. Thus, *M. fermentans* may induce necrotic cell death via TLR4 signaling to upregulate IFITM3 for Aβ_1–42_ deposition. In contrast to human neuronal cells, murine *IFITM3* gene expression was not upregulated by *M. fermentans* (Supplementary Fig. S6a). This could be due to sequence differences in the ligand binding sites (560 of 843 amino acids, 66%) between murine and human TLR4 (Supplementary Fig. S6b). These results suggest that *M. fermentans* infection in human neuronal cells induces TLR4 signaling to increase Aβ_1−42_ accumulation via an IFITM3-mediated pathway, resulting in necrotic cell death. To determine whether the observed effects are specific to *M. fermentans* infection or are a general response to TLR4-activating ligands, we performed additional experiments. We added CRX-527, a CD14-independent TLR4 agonist^37^, and α-enolase, a protein expressed on the cell surface of *M. fermentans* that can activate the CD14-dependent TLR4 signaling pathway^38–40^, to SH-SY5Y cells. The results showed that neither CRX-527 nor α-enolase alone induced cell death, upregulated h*TLR4,* h*IL-6,* h*TNF-α,* h*IL-1β,* or h*IFITM3* mRNA levels, or increased intracellular Aβ_1–42_ in SH-SY5Y cells (Supplementary Fig. S7a–e). These results are in line with those of previous research showing no impact of lipopolysaccharide (LPS) on SH-SY5Y cells^41–45^. Thus, the consequences of *M. fermentans* infection, such as Aβ_1–42_ deposition and cell death, would appear to be specifically linked to TLR4 upregulation. We also found no increase in cell death of SH-SY5Y cells after adding the α-enolase to *M. fermentans*-infected cells (Supplementary Fig. S7f) while an TLR4 antagonist blocked IFITM3 upregulation and necrotic cell death. These findings suggest that *M. fermentans* is sufficient for the activation of TLR-4 because α-enolase did not increase necrotic cell death.

## Discussion

*M. fermentans* infection is associated with various neurological diseases such as CFS, GWS, ALS, and ASD^5–7,20–24^. This suggests that *M. fermentans* infection could have pathological relevance for neurological changes associated with shared symptoms in CFS, and GWS, ALS, and ASD, such as cognitive and neurological problems^46^. Indeed, mycoplasmas have been isolated from the brains of various types of animals, suggesting they can directly invade the CNS^8,9,11,15,16,47–49^. Additionally, *M. fermentans* have been detected in numerous tissues including the brains of AIDS patients^9,10^, and was also detected in a case study of non-AIDS patients with acute fatal disease^13,14^. Thus, *M. fermentans* may be an underestimated pathogen in neurological diseases. We therefore investigated pathological changes in neuronal cells infected with *M. fermentans*. Surprisingly, *M. fermentans* could infect human neuronal cells, in which it replicated and induced necrotic cell death, but it could not induce necrotic cell death in mouse neuronal cells.

IFITM3, a member of the interferon-induced transmembrane protein family, is a restriction factor that prevents enveloped viral particles from entering host cells by blocking membrane fusion during endocytosis^50,51^. It also blocks mycobacterial growth^35^, plays a critical role in intrinsic antiviral immunity of human cells, and restricts cell entry of diverse viruses including influenza virus, West Nile virus, dengue virus, Ebola virus, and coronavirus^52^. IFITMs are potent antiviral effectors known for their ability to inhibit fusion between viral and cellular membranes, and they are widely studied for their potential as antiviral therapies^51,52^. Interestingly, a study revealed that IFITM3 in neurons can bind to γ-secretase to upregulate its activity, thereby increasing the production of Aβ_1–42_^34^. In our current study, we also found that *M. fermentans* could upregulate IFITM3, which led to deposition of Aβ_1–42_. Eventually, this triggered necrotic cell death in infected human neuronal cells. Thus, our results suggest that *M. fermentans* may contribute to deposition of Aβ in the CNS of *M. fermentans*-infected patients. Analysis of the mechanism showed that a TLR4 antagonist reduced *IFITM3* gene expression and necrotic cell death in *M. fermentans*-infected SH-SY5Y cells, suggesting that TLR4 is involved in IFITM3 upregulation in *M. fermentans* infection. Although mycoplasmas do not possess LPS, *M. fermentans* cells express α-enolase, which can activate the TLR4 signaling pathway on their cell surfaces^38–40^. However, we observed that neither α-enolase nor the TLR4 agonist CRX-527 alone induced cell death or activated the TLR4 signaling pathway in SH-SY5Y cells. This finding is consistent with previous studies showing that direct application of LPS, a TLR4 agonist, has no effect on SH-SY5Y cell death^43–45^. Unlike other cells, SH-SY5Y cells do not exhibit cytokine transcription or release in response to LPS, possibly due to their low levels of TLR4 expression^41,42^. Thus, our results suggest that *M. fermentans* infection-mediated additional processes are required for necrotic cell death. In addition, addition of α-enolase did not enhance the cell death effect beyond that observed with *M. fermentans* infection alone, whereas the TLR4 antagonist blocked cell death. This also suggests that *M. fermentans* infection is sufficient for TLR4 activation and induction of cell death.

In our experiments, we tested two mouse neuronal cell lines, neuro2a and HT-22, but neither of these cell lines exhibited *IFITM3* upregulation or necrotic cell death after *M. fermentans* infection. However, we observed necrotic cell death of the human SH-SY5Y neuronal cell line. In addition, this effect by *M. fermentans* infection was observed in organoid cultures, minimizing the concern about extrapolating from a pure cell line approach. Thus, our results may indicate that *M. fermentans*-induced immunological responses to pathogen infection can differ depending on the species, as shown in a previous study^53^. Overall, our findings suggest that intracellular Aβ_1–42_ deposition by TLR4-mediated IFITM3 upregulation is the main inducer of necrotic cell death.

The amphipathic helix of IFITM3 is essential for its antiviral activity by inducing membrane deformation in virus-infected cells^54^. However, a previous study revealed that IFITM3 also plays a modulatory role in γ-secretase activity by binding to PS1 near the active site, resulting in an increase in γ-secretase activity and Aβ production^34^. Thus, it is essential to consider the potential antiviral effects of any therapeutic targeting of IFITM3 since the determinants involved in its antiviral activity may also play a role in its interaction with γ-secretase and regulation of Aβ production. It is worth noting that oligomerization of IFITM3 is necessary for its antiviral activity through the induction of membrane stiffening^55^, whereas IFITM3 binds to PS1-NTF as a monomer or dimer^34^. This suggests that the antiviral mechanism of IFITM3 and its role in modulating γ-secretase activity may involve distinct determinants. Therefore, further investigations are necessary to fully comprehend the determinants of IFITM3 involved in this complex and their impact on γ-secretase activity, which could have significant implications for the development of targeted therapies that balance its antiviral and modulatory effects.

In our study, we observed a significant downregulation of *IL-1β* and *PRNP* gene expression in *M. fermentans*-infected SH-SY5Y cells following h*APP* knockdown. This suggests that these genes may function downstream of the signaling pathway leading to Aβ_1–42_ deposition in our experimental system, which is consistent with previous studies implicating IL-1β and PRNP in Aβ-induced neuroinflammation and neurotoxicity^26,28,31,33^. Interestingly, we also observed upregulation of *APOE* expression following h*APP* knockdown in *M. fermentans*-infected SH-SY5Y cells. APOE has been implicated in AD pathology, where it plays a complex role in the clearance and aggregation of Aβ in an isoform-specific manner^32^. Therefore, this finding may reflect a compensatory mechanism that counteracts Aβ-induced neurotoxicity. However, the exact role of the Aβ pathway in human PRNP or APOE remains unclear. Therefore, it is important to note that the observed effects of h*APP* knockdown on gene expression may be indirect, and further studies are needed to confirm their precise positioning within the signaling pathway leading to Aβ deposition. Nonetheless, our findings provide important insights into the potential roles of IL-1β, PRNP, and APOE in this process, and suggest new avenues for future research.

*M. fermentans* has been found in various tissues of AIDS patients, leading to its designation as an opportunistic pathogen^4^. Interestingly, several studies showed that *M. fermentans* is present in saliva in about half of the population (110 of 201, 54.7%; 49 of 110, 44%), suggesting that the organism colonizes the human mouth and is transmitted easily to others^56,57^. However, blood infection did not occur frequently in healthy individuals^5–7,20–24^. It is possible that the organism invades blood and various tissues including the brain in immunocompromised individuals, altering physiological conditions and causing *M. fermentans*-related neurological diseases. Thus, our results suggest the possibility that opportunistic CNS infection by *M. fermentans* may occur in immune-altered individuals, resulting in other types of neurological diseases.

In conclusion, in vitro and ex vivo analyses revealed that *M. fermentans* can function in a pathogenic capacity in human neuronal cells. Cellular and molecular analyses of *M. fermentans* pathogenicity revealed that *M. fermentans* directly induces necrotic neuronal cell death by IFITM3-mediataed Aβ_1–42_ deposition. Thus, our results suggest that *M. fermentans* is involved in the pathogenicity of neurological diseases. Furthermore, our findings could help expand our understanding of neurological diseases caused by mycoplasmal infections.

## Methods

### Cell lines

SH-SY5Y cells from the Korean Cell Line Bank (KCLB, Seoul, Republic of Korea, Cat. No. 22266), HT-22 cells (gifted from Dr. Inhee Mook-Jung, Seoul National University), and neuro2a cells from the American Type Culture Collection (ATCC, Manassas, VA, USA, Cat. No. CCL-131) were cultured in 10% fetal bovine serum (FBS, Hyclone, Logan, UT, USA, Cat. No. SH3008403), 100 U/ml penicillin, and 100 μg/ml streptomycin-supplemented Dulbecco’s modified Eagle’s medium (DMEM, Hyclone, Cat. No. SH30243.01) at 37 °C with 5% CO2. Human induced pluripotent stem cells (IPSCs) CMC-hiPSC-003 and CMC-hiPSC-009 were provided by the National Stem Cell Bank of Korea (Korea National Institute of Health), originally provided from Catholic University. IPSCs were cultured using an mTeSR 1 Complete Kit (StemCell Technologies, Vancouver, BC, Canada, Cat. No. 85850). Control-, h*APP*-, and h*IFITM3*-knockdown SH-SY5Y cells were established via infection of control shRNA lentivirus particle-A (Santa Cruz Biotechnology, Dallas, TX, USA, Cat. No. sc-108080), hAPP shRNA lentivirus particles (Santa Cruz Biotechnology, Cat. No. sc-29677-V), and hIFITM3 shRNA lentivirus particles (Santa Cruz Biotechnology, Cat. No. sc-97053-V). Knockdown SH-SY5Y cells were maintained under 5 μg/ml puromycin dihydrochloride (Sigma-Aldrich, St. Louis, MO, USA, Cat. No. P8833-10 mg).

### Preparation and infection of *M. fermentans*

*M. fermentans* strain PG-18 (ATCC, Cat. No. ATCC 19989) was used in this study. *M. fermentans* strains were grown in ATCC mycoplasma medium (ATCC medium 243) under anaerobic conditions using an anaerobic jar and indicator. We followed the instructions suggested for culturing Mollicutes from ATCC. The number of viable mycoplasma cells was determined by plating and was calculated as the number of colony-forming units (CFU) per milliliter under anaerobic conditions. For infection of *M. fermentans,* SH-SY5Y cells were seeded into 3 ml culture medium in a 6-well cell culture plate at a density of 5 × 10^5^ cells per well, while HT-22 cells were seeded at 0.5 × 10^5^ cells per well, and neuro2a cells were seeded at 1 × 10^5^ cells per well. We infected these cells with *M. fermentans* at MOI = 0.01–1 and confirmed that necrotic cell death of SH-SY5Y cells occurred at earlier time points at higher MOI. Therefore, we used MOI = 0.01 throughout for the in vitro infection system. Before *M. fermentans* infection, we centrifuged *M. fermentans* cells at 26,000 g for 30 min at 4 °C and replaced mycoplasma medium with 1 ml of cell culture medium, and repeated this twice. After 24 h of infection, we replaced the cell culture medium. In the case of brain organoids, Matrigel (Corning, Corning, NY, USA, Cat. No. 354277) around organoids could inhibit the infection, so we increased the MOI to 0.05. We estimated the number of cells in one brain organoid to be ~ 1 × 10^5^ cells, and we replaced the medium after 72 h of infection. Inactivated *M. fermentans* particles were prepared by preincubation with 70% EtOH for 10 min, heating at 56 °C for 30 min, and exposure to UV irradiation (253.7 nm) for 10 min. For mycoplasma antibiotic drug testing, we used *M. fermentans* pretreated with 1 mg/ml Primocin (InvivoGen, San Diego, CA, USA, Cat. No. ant-pm-05) and 125 μg/ml Myco-Guard Mycoplasma Elimination Reagent (Biomaxinc, Seoul, Republic of Korea, Cat. No. SMD022) for 3 h at 37 °C, then diluted 1:10 in culture medium before infection.

### Annexin V/PI apoptosis assay

To confirm the mechanism of cell death, we performed annexin V/PI apoptosis assays using an Annexin V Apoptosis Detection Kit APC (eBioscience, San Diego, CA, USA, Cat. No. 88-8007-74) according to the manufacturer’s instructions, and analyzed the results on a Guava EasyCyte HT instrument (Merck Millipore, Burlington, MA, USA) or a BD FACSCanto II (Becton, Dickinson and Company, Franklin Lakes, NJ, USA), then analyzed with FlowJo v10.7.1 (Becton, Dickinson and Company) data analysis software.

### Differentiation of SH-SY5Y cells and preparation of conditioned medium

To induce differentiated SH-SY5Y cells, they were seeded into 3 ml of medium at a density of 2 × 10^5^ cells per a well in a 6-well cell culture plate. After 24 h, cells were treated with 80 mM PMA (Sigma-Aldrich, Cat. No. P1585-1MG) for 7 days. Infection was performed at an MOI of 0.01, and medium was replaced with fresh medium after 24 h. At 7, 12, and 19 dpi, cells were used for analysis. At 19 dpi, the cell culture medium was replaced with fresh medium, and after 2 days of incubation, the conditioned medium was harvested and centrifuged at 900 × g at 4 °C for 30 min, and supernatants were passed through a 0.45 μm pore size membrane filter. The conditioned medium was then diluted 1:3 in fresh culture medium and used to treat fresh differentiated SH-SY5Y cells.

### Quantification of *M. fermentans* DNA levels

For quantification of intracellular or secreted *M. fermentans* DNA levels, total DNA was extracted using a G-spin Total DNA Extraction Mini Kit (iNtRON Biotechnology, Gyeonggi-do, Republic of Korea, Cat. No. TCS0803). qPCR was then performed using SYBR Green qPCR 2 × Premix (Enzynomics) on a CFX Connect Real-Time PCR Detection System (Bio-Rad). Intracellular *M. fermentans* DNA levels were normalized against internal control DNA levels. qPCR primer sequences were as follows: *M. fermentans*-F, 5′-GGACTATTGTCTAAACAATTTCCC-3′; *M. fermentans*-R, 5′-GGTTATTCGATTTCTAAATCGCCT-3′; mouse internal control-F, 5′-CTGTGGATGGCCCCTCTG-3′; mouse internal control-R, 5′-CACGACGGACACATTGGG-3′; human internal control-F, 5′- GGCTGTTGTCATACTTCTCATG-3′; human internal control-R, 5′- GGACCAAGATCCCTCCAAAAT-3′.

### Quantification of gene expression levels

To quantify gene expression, total cellular RNA was isolated using an RNeasy Mini Kit (Qiagen, Hilden, Germany, Cat. No. 74104), and cDNA was prepared using TOPscript RT Drymix (Enzynomics, Daejeon, Republic of Korea, Cat. No. RT100). qRT-PCR was then performed using SYBR Green qPCR 2 × Premix (Enzynomics, Cat. No. RT500-M) on a CFX Connect Real-Time PCR Detection System (Bio-Rad, Hercules, CA, USA). Expression levels of each gene were normalized against glyceraldehyde-3-phosphate dehydrogenase (*GAPDH*) gene expression levels. qRT-PCR primer sequences were as follows: h*GAPDH*-F, 5′-GGAGCGAGATCCCTCCAAAAT-3′; h*GAPDH*-R, 5′-GGCTGTTGTCATACTTCTCATG-3′; h*APP*-F, 5′-GCTGGTGGAGACACACATG-3′; h*APP*-R, 5′-GGATCTGAGCGGCTTTCTTG-3′; h*MAP2*-F, 5′-TGCGCCCAGATTTTATTGATC-3′; h*MAP2*-R, 5′-GTTCGTTGTGTCGTGTTCTCA-3′; h*APOE-*F, 5′-GGGTCGCTTTTGGGATTACCTG-3′; h*APOE*-R, 5′-CAACTCCTTCATGGTCTCGTCC-3′; h*IL-1β*-F, 5′-CCACAGACCTTCCAGGAGAATG-3′; h*IL-1β*-R, 5′-GTGCAGTTCAGTGATCGTACAGG-3′; h*TNF*-α-F, 5′-ACGCTCTTCTGCCTGCTG-3′; h*TNF-α*-R, 5′-GCTTGAGGGTTTGCTACAACA-3′; h*PRNP*-F, 5′-CTGCTGGATGCTGGTTCTCT-3′; h*PRNP*-R, 5′-GTGTTCCATCCTCCAGGCTT-3′; h*IFITM3*-F, 5′-GGTCTTCGCTGGACACCAT-3′, h*IFITM3*-R, 5′-TGTCCCTAGACTTCACGGAGTA-3′; m*GAPDH*-F, 5′-GCACAGTCAAGGCCGAGAAT-3′; m*GAPDH*-R, 5′-GCCTTCTCCATGGTGGTGAA-3′; h*TLR4*-F, 5′-GACTTGCGGGTTCTACATCA-3′; h*TLR4*-R, 5′-CATAGGGTTCAGGGACAGGT-3′. h*IL-6*-F, 5′-TGTGAAAGCAGCAAAGAGGC-3′; h*IL-6*-R, 5′-CCAGGCAAGTCTCCTCATTG-3′.

### Western blotting analysis

Proteins were separated by sodium dodecyl sulfate polyacrylamide gel electrophoresis (SDS-PAGE) using 12–15% gels and transferred to a polyvinylidene difluoride membrane. Membranes were then incubated with the following primary antibodies: β-Amyloid (1–42 Specific, D9A3A, Cell Signaling Technology, Danvers, MA, USA, Cat. No. 14974S), Tau (HT7, Thermo Fisher Scientific, Waltham, MA, USA, Cat. No. MN1000), Phospho-Tau (Ser202, Thr205, AT8, Thermo Fisher Scientific, Cat. No. MN1020), GAPDH (H-12, Santa Cruz Biotechnology, Cat. No. sc-166574), and IFITM3 (F-41, Santa Cruz Biotechnology, Cat. No. sc-100768). Peroxidase-conjugated anti-rabbit and anti-mouse secondary antibodies (Sigma-Aldrich, Cat. No. A0545 and A2554) for ECL substrate (GE Healthcare, Barrington, IL, USA) were then employed. To check the quality of p-tau bands, we used CIP at 10 and 40 units (New England Biolabs, Ipswich, MA, USA, Cat. No. M0290) for 60 min at 37 °C. It is important to note that the original blots were cut before hybridization with antibodies. For this reason, full-length blots cannot be provided. The images in Supplementary Fig. S9 depict the blots after cropping.

### Immunocytochemistry and immunohistochemistry analysis

For immunocytochemical staining, cells were fixed with 2% paraformaldehyde (PFA) for 20 min and permeabilized with 0.3% Triton X-100 in phosphate-buffered saline (PBS) for 20 min, and then blocked with 0.5% bovine serum albumin (BSA) in PBS for 1 h at room temperature (RT). Cells were stained with primary antibodies in 0.5% BSA in PBS for 2 h at RT, and then stained with Alexa Fluor 488-conjugated anti-mouse IgG antibody and 594-conjugated anti-rabbit IgG antibody (Thermo Fisher Scientific, Cat. No. A-11029 and A-11037) in 0.5% BSA in PBS for 45 min at RT. Images were captured with an FV1000 confocal microscope (Olympus, Tokyo, Japan). Quantification analyses were performed using ImageJ software (National Institutes of Health, Bethesda, MD, USA; https://imagej.nih.gov/ij/features.html).

For immunohistochemical staining, brain organoids were embedded in optimal cutting temperature (OCT) compound after fixation with 4% PFA for 24 h at 4 °C and submerged in 30% sucrose. After slicing the OCT-embedded frozen block into 12 μm sections, brain organoids were permeabilized with 0.3% Triton X-100 in PBS for 20 min and blocked with 1% BSA in PBS for 1 h, and tissues were stained with primary antibodies in 0.5% BSA in PBS for 24 h at 4 °C, followed by Alexa Fluor 488-conjugated anti-mouse IgG antibody and 594-conjugated anti-rabbit IgG antibody (Thermo Fisher Scientific). Images were captured with a Leica TCS SP8 confocal microscope (Leica Microsystems, Wetzlar, Germany). Analyses were performed using Leica Application Suite X software (Leica Microsystems). The following primary antibodies were used: SOX2 (D6D9, Cell Signaling Technology, Cat. No. C3579S), Tubulin β 3 (TUBB3, BioLegend, Cat. No. 801201), β-Amyloid (1–42 Specific, D9A3A, Cell Signaling Technology, Cat. No. 14974S), Phospho-Tau (Ser202, Thr205, AT8, Thermo Fisher Scientific, Cat. No. MN1020), Phospho-MLKL (Ser358, Cell Signaling Technology, Cat. No. D6H3V), and β-actin (ABclonal, Woburn, MA, USA, Cat. No. AC026). Nuclei were visualized by staining with 20 μg/ml of Hoechst 33342 (Sigma-Aldrich, Cat. No. B2261-25MG) in PBS.

### Generation of brain organoids

To generate brain organoids, we used a STEMdiff Cerebral Organoid Kit (StemCell Technologies, Cat. No. 08570) and a Cerebral Organoid Maturation Kit (StemCell Technologies, Cat. No. 08571) according to the manufacturer’s protocols. Briefly, to stimulate embryoid body (EB) formation, iPSCs were dissociated with ReLeSR (StemCell Technologies, Cat. No. 05872) and seeded in 100 μl of EB seeding medium containing 10 μM Y-27632 (StemCell Technologies, Cat. No. 72302) at 9,000 cells per well in a 96-well round-bottom ultralow attachment plate. On days 2 and 4, 100 μl of EB formation medium was added. On day 5, EBs of 400–600 μm in size with round and smooth edges were used for induction. Briefly, one or two EBs were incubated in 500 μl of induction medium per well in a 24-well ultralow attachment plate. On day 7, EBs were embedded in Matrigel (Corning), and 12–16 embedded EBs were incubated in 3 ml of expansion medium per well in a 6-well ultralow adherent plate. On day 10, brain organoids were matured in maturation medium on an orbital shaker at 65 rpm, and maturation medium was changed every 3 days.

### RNA-seq analysis

For RNA-seq, libraries were prepared using a TruSeq Stranded Total RNA LT Sample Prep Kit (Illumina, San Diego, CA, USA) according to the TruSeq stranded total RNA samples prep guide. Sequencing was performed with a NovaSeq 6000 system (Illumina). Genes with a fold change ≥ 2 and transcripts per million (TPM) ≥ 1 were defined as differentially expressed genes and displayed in a scatter plot. Gene functional classification was performed using the DAVID (release 6.8; https://david.ncifcrf.gov). EASE scores, a modified Fisher Exact p-value, (*p* ≤ 0.05) were used to identify gene enrichment of functional pathways by estimating the number of genes belonging to functional pathways involving the selected target proteins.

### Enzyme-linked immunosorbent assay (ELISA)

For quantification of secreted Aβ_1–42_ in culture medium, we used a commercial ELISA Kit for Amyloid Beta Peptide 1–42 (Wuhan USCN Business Co., Ltd., Wuhan, China, Cat. No. CEA946Hu) according to the manufacturer’s protocols.

### Treatments with neutralizing antibody, antagonist, and ligands

We used 50 μM human TLR2/1 and TLR2/6 and murine TLR2/1 signaling inhibitor (InvivoGen, Cat. No. inh-c29) as a TLR2 antagonist, and 10 μg/ml of ultrapure lipopolysaccharide from *Rhodobacter sphaeroides* (InvivoGen, Cat. No. tlrl-prslps) as a TLR4 antagonist. For IL-1β neutralization, we used 1 μg/ml of human IL-1β neutralizing antibody (4H5, InvivoGen, Cat. No. mabg-hil1b-3), and 100 ng/ml TNF-α neutralizing antibody (SinoBiological Inc. Beijing, China, Cat. No. 10602-MM0N1) was used for TNF-α neutralization. CRX-527 (InvivoGen, Cat. No. tlrl-crx527) at 1 μg/ml was used as an CD14-independent TLR4 agonist^37^. Additionally, 10 μg/ml of active α-enolase protein (Mybiosource, Vancouver, Canada, Cat. No. MBS203689) was added as an activator of the CD14-dependent TLR4 signaling pathway^40^. SH-SY5Y cells were pretreated with these inhibitors for 1 h before *M. fermentans* infection at 0.1 MOI. After 48 h, we replaced the medium with fresh medium containing inhibitors. At 3 dpi we analyzed the *M. fermentans*-infected cells.

### Statistical analysis

The significance of differences between two groups was analyzed using unpaired Student’s t-tests using Origin2021 software (OriginLab Corporation, Northampton, MA, USA). Quantification of protein expression levels in western blotting was performed using ImageJ software, GAPDH served as an internal control for normalization, and Hoechst was used for normalization of immunocytochemical data. All results are expressed as the mean ± standard deviation (SD). For all statistical tests, *p*-values ≤ 0.05 were considered significant.

## Supplementary Information

Supplementary Information.

## Data Availability

The original contributions presented in the study are included in the article/Supplementary Material. Further inquiries can be directed to the corresponding author. All RNA-seq data generated in this study are available through the NCBI Sequence Read Archive (SRA) through accession numbers SRR22252649-SRR22252651 under BioProject PRJNA900032.
