# Supplementary material for: Mycoplasma fermentans infection induces human necrotic neuronal cell death via IFITM3-mediated amyloid-β (1–42) deposition
Source: Sci Rep. 2023 Apr 26;13:6864. doi: 10.1038/s41598-023-34105-y (PMC10132800; doi:10.1038/s41598-023-34105-y)
Supplement: Supplementary file 1 — Supplementary Information. [file 41598_2023_34105_MOESM1_ESM.pdf]

Supplementary Information for

Mycoplasma fermentans infection induces human necrotic neuronal cell death via IFITM3-mediated amyloid-β (1-42) deposition

Kyu-Young Sim[1,2], Yeongseon Byeon[2], So-Eun Bae[1], Taewoo Yang[1,2], Cho-Rong Lee[1], Sung-Gyoo Park[1,*]

[1]Institute of Pharmaceutical Sciences, College of Pharmacy, Seoul National University, Seoul, Republic of Korea
[2]School of Life Sciences, Gwangju Institute of Science and Technology (GIST), Gwangju, Republic of Korea

***Correspondence:** Sung-Gyoo Park
Email: riceo2@snu.ac.kr

This PDF file includes:

Supplementary methods
Supplementary Figures S1 to S9
Supplementary Table S1
Supplementary references

Supplementary methods

Analysis of the Odds ratio (OR)

To summarize the analysis of the OR of each clinical cases for these diseases, we used the OR calculator (Version 20.110; MedCalc Software Ltd, Ostend, Belgium; https://www.medcalc.org/calc/odds_ratio.php). OR values were calculated using the equation $\mathrm{OR} = \frac{a/b}{c/d} = \frac{a \times d}{b \times c}$, the standard error of log OR values was calculated using the formula $\mathrm{SE}\{\ln(\mathrm{OR})\} = \sqrt{\frac{1}{a} + \frac{1}{b} + \frac{1}{c} + \frac{1}{d}}$, and the 95% confidence interval was calculated using the expression $95\%\ \mathrm{CI} = \exp(\ln(\mathrm{OR}) - 1.96 \times \mathrm{SE}\{\ln(\mathrm{OR})\})$ to $\exp(\ln(\mathrm{OR}) + 1.96 \times \mathrm{SE}\{\ln(\mathrm{OR})\})$.

Amino acid sequence alignment

Alignment of human TLR4 (NCBI Reference Sequence: NP_612564.1) and mouse TLR4 (NCBI Reference Sequence: NP_067272.1) amino acid sequences was conducted with pairwise sequence alignment tools based on the EMBOSS Needle using the Needleman-Wunsch algorithm (EMBL's European Bioinformatics Institute, Hinxton, Cambridgeshire, UK; https://www.ebi.ac.uk/Tools/psa/emboss_needle/).

Flow cytometry

For intracellular staining, cells were fixed with 2% paraformaldehyde (PFA) for 20 min and permeabilized with 0.3% Triton X-100 in phosphate-buffered saline (PBS) for 20 min, and then blocked with 0.5% bovine serum albumin (BSA) in PBS for 1 h at room temperature (RT). Cells were stained with primary antibody for β-Amyloid (1–42 Specific, D9A3A, Cell Signaling Technology, Danvers, MA, USA, Cat. No. 14974S) in 0.5% BSA in PBS for 2 h at RT, and then stained with 594-conjugated anti-rabbit IgG antibody (Thermo Fisher Scientific, Cat. No. A-11037) or R-phycoerythrin conjugated anti-rabbit IgG (Jackson ImmunoResearch, Cat. No. 111-117-008) in 0.5% BSA in PBS for 45 min at RT. We analyzed the results on a Guava EasyCyte HT instrument (Merck Millipore, Burlington, MA, USA) or a BD FACS Canto II (Becton, Dickinson and Company, Franklin Lakes, NJ, United States), then analyzed with FlowJo v10.7.1 (Becton, Dickinson and Company) data analysis software.

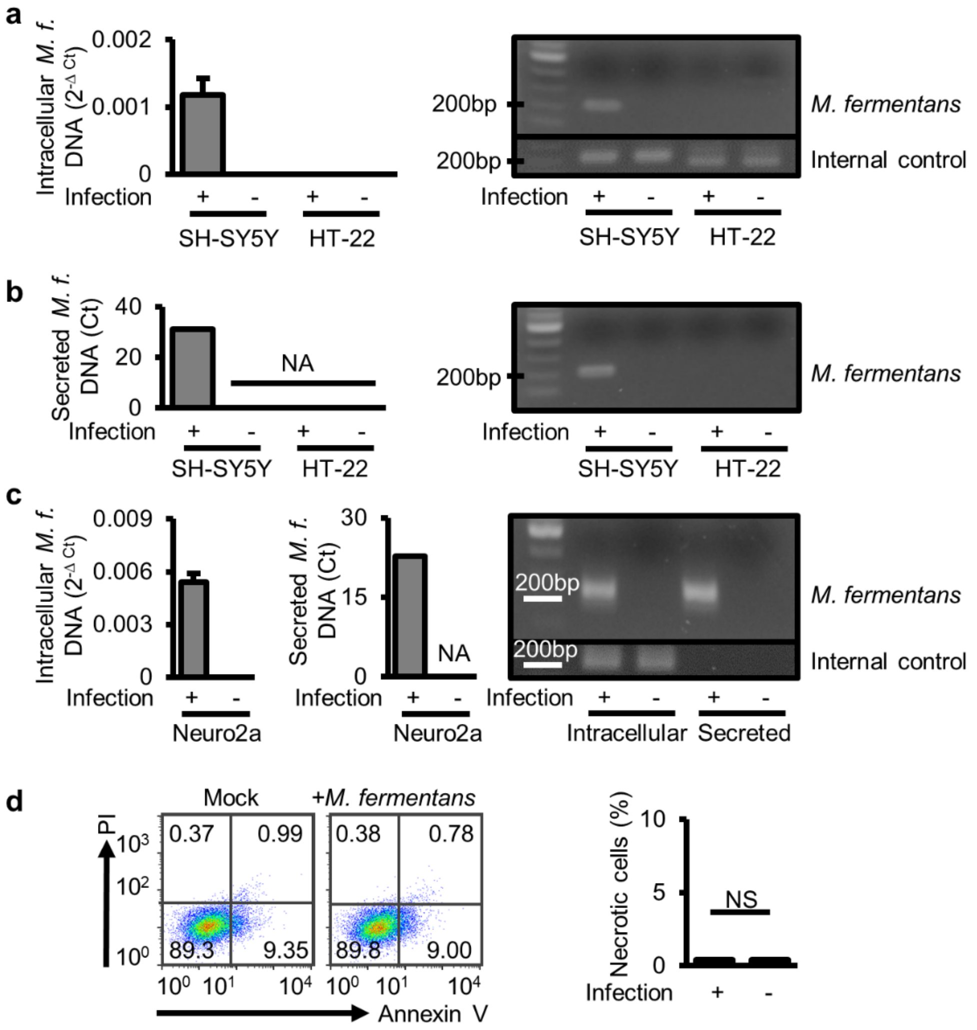

Supplementary Figure S1. ***M. fermentans*** **replicates in some neuronal cells, but it does not induce necrotic cell death in mouse neuronal cells.** (**a**) Bar graph of intracellular *M. fermentans* DNA levels normalized against an internal control determined by qPCR in SH-SY5Y cells and HT-22 cells at 12 dpi, and representative agarose gel electrophoresis image of fragments amplified using the indicated primers. (**b**) Bar graph of secreted *M. fermentans* DNA levels determined by qPCR in the culture medium of SH-SY5Y cells and HT-22 cells at 12 dpi, and representative agarose gel electrophoresis image of fragments amplified using the indicated primers. (**c**) Bar graph of *M. fermentans* intracellular and secreted DNA copies in neuro2a cells at 12 dpi, and representative agarose gel electrophoresis image of fragments amplified using the indicated primers. (**d**) Representative flow cytometry dot plots from Annexin V/PI apoptosis assays for neuro2a cells at 12 dpi, and a bar graph of Annexin V-negative PI-positive cells from (**d**). Bar graphs present mean values ± SD. M. f., *Mycoplasma fermentans*; Ct, cycle threshold; NA, not applicable; NS, not significant (unpaired Student's t-test). Data are averages from three or more independent experiments. Original gel images are presented in Supplementary Figure S8.

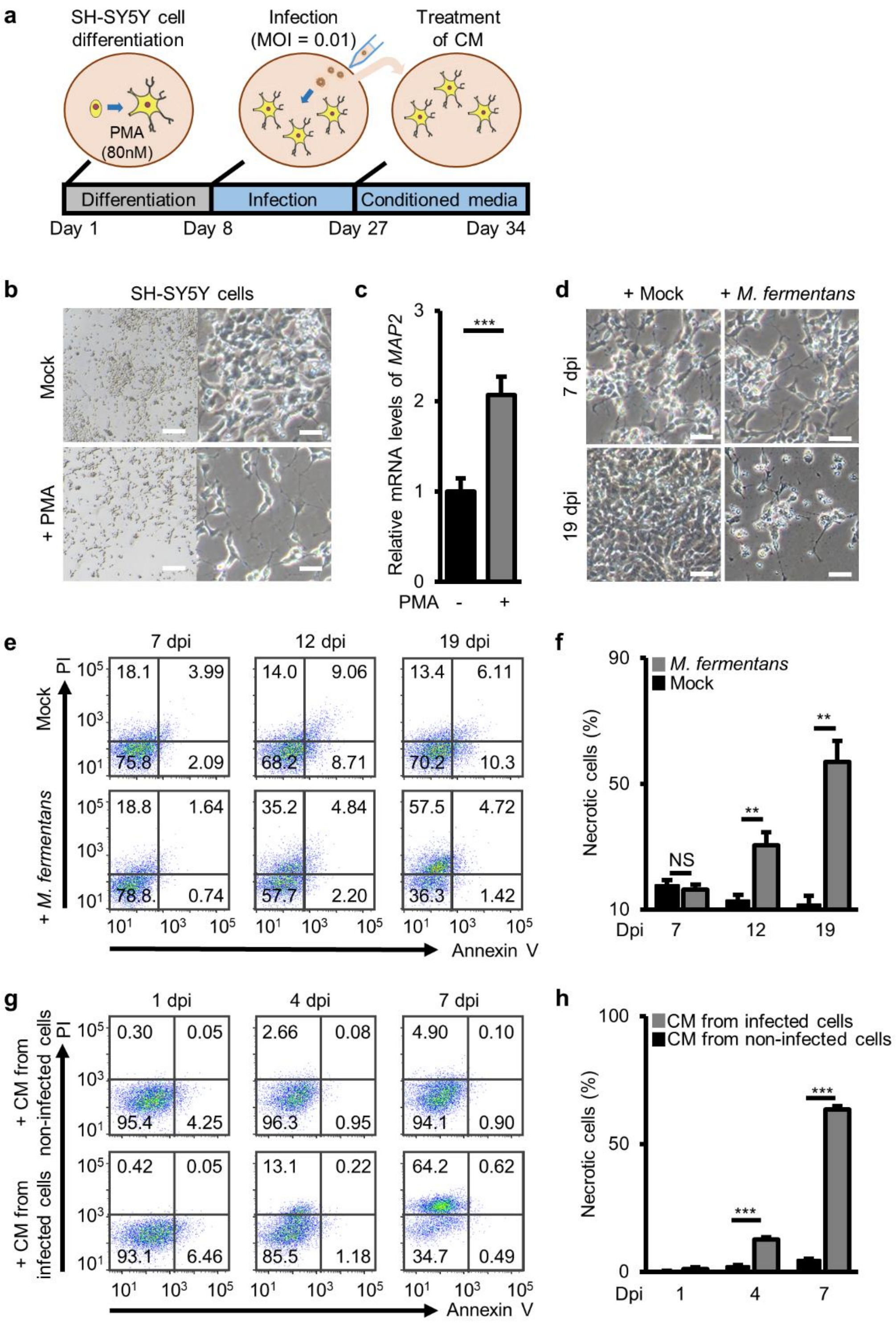

Supplementary Figure S2. ***M. fermentans*** **induces necrotic cell death in differentiated SH-SY5Y cells.** (**a**) Schematic diagram of *M. fermentans* infection of differentiated SH-SY5Y cells, and infectivity tests using conditioned medium (CM). (**b**) Representative microscopy images of undifferentiated and differentiated SH-SY5Y cells at 7 days post-treatment for mock and PMA (80 nM) groups. (**c**) Bar graph of h*MAP2* gene expression

levels normalized against *GAPDH* in PMA-induced SH-SY5Y cells relative to mock-induced SH-SH5Y cells, determined by qPCR. (**d**) Representative images of *M. fermentans*-infected differentiated SH-SY5Y cells at 7 and 19 dpi. (**e**) Representative flow cytometry dot plots from Annexin V/PI apoptosis assays for differentiated SH-SY5Y cells at 7, 12, and 19 dpi. (**f**) Bar graph of Annexin V-negative PI-positive cells from (**e**). (**g**) Representative flow cytometry dot plots from Annexin V/PI apoptosis assays for differentiated SH-SY5Y cells at 1, 4, and 7 dpi for CM derived from *M. fermentans*-infected or non-infected differentiated SH-SY5Y cells. (**h**) Bar graph of Annexin V-negative PI-positive cells for data from (**g**). Scale bars = 500 μm for images on the left and 100 μm images on the right for (**b, d**). Bar graphs present mean values ± SD; $**p \leq 0.01$; $***p \leq 0.001$; NS, not significant (unpaired Student's t-test); M. f., *Mycoplasma fermentans*; Ct, cycle threshold; NA, not applicable. Data. Data are averages of three or more independent experiments.

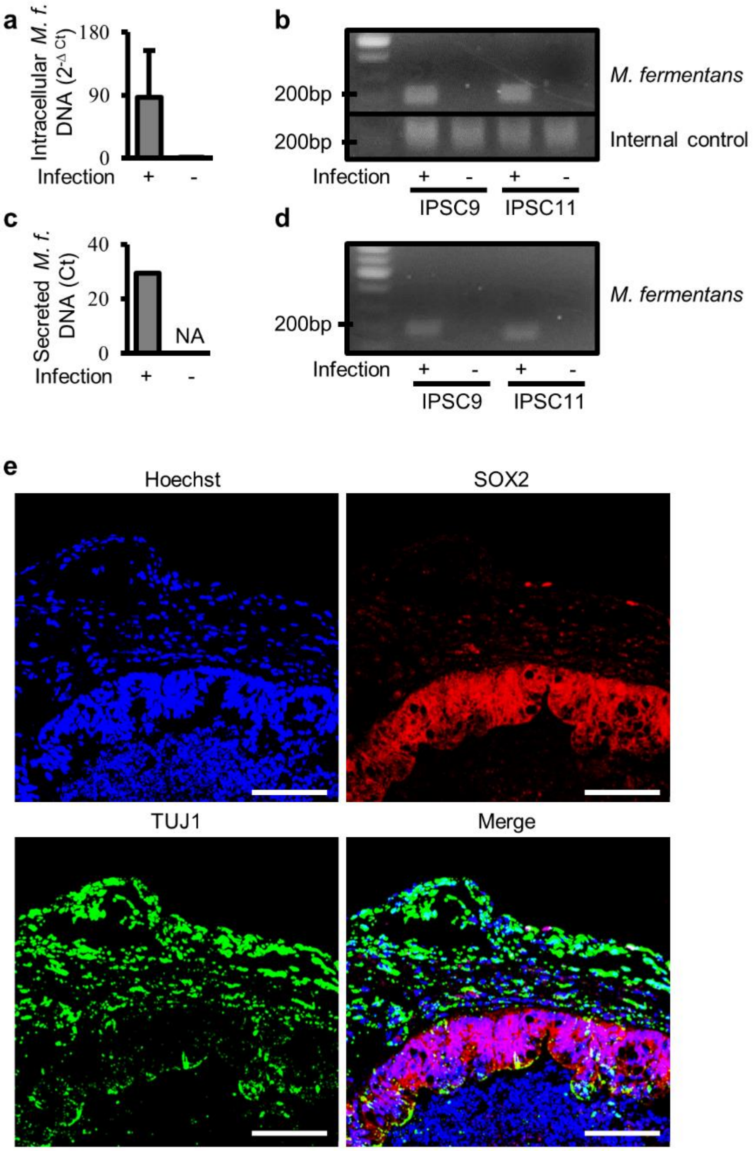

Supplementary Figure S3. ***M. fermentans*** **infection in human brain organoids derived from IPSC cells.** (**a**) Bar graph of intracellular *M. fermentans* DNA levels normalized against internal controls determined by qPCR in mock- or *M. fermentans*-infected brain organoids at 26 dpi. (**b**) Representative agarose gel electrophoresis image of fragments amplified using the indicated primers to analyze data from (**a**). (**c**) Bar graph of secreted *M. fermentans* DNA levels in the culture medium of mock- or *M. fermentans*-infected brain organoids determined by qPCR at 26 dpi. (**d**) Representative agarose gel electrophoresis image of fragments amplified using the indicated primers to analyze data from (**c**). (**e**) Representative immunohistochemistry images of brain organoids containing mature neurons (TUJ1, green) and neural progenitor cells (SOX2, red) at day 67 of differentiation. Scale bars = 100 μm for images. M. f., *Mycoplasma fermentans*; Ct, cycle threshold; NA, not applicable. Data are averages from three or more independent experiments. Original gel images are presented in Supplementary Figure S8.

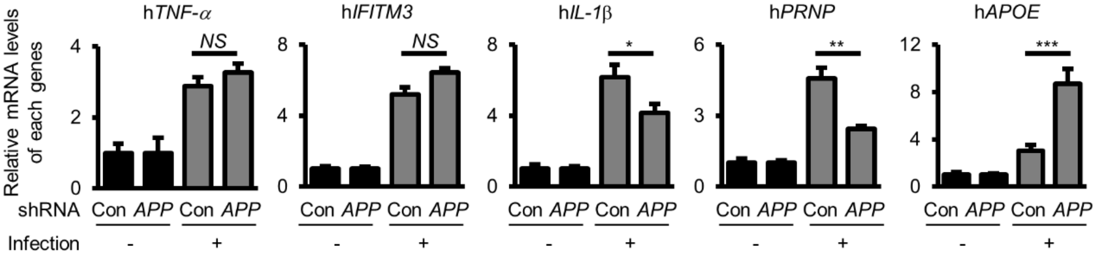

Supplementary Figure S4. Knockdown of h*APP* modulates expression of genes associated with necrotic cell death induced by *M. fermentans* infection in SH-SY5Y cells. Bar graph of indicated gene expression levels normalized against the *GAPDH* gene in *M. fermentans*-infected SH-SY5Y cells with indicated gene knockdown relative to mock-infected SH-SY5Y cells with indicated gene knockdown at 12 dpi, determined by qPCR. Con, control knockdown SH-SY5Y cells; *APP*, h*APP* knockdown SH-SY5Y cells. Bar graphs present mean values ± SD. $*p \leq 0.05$; $**p \leq 0.01$; $***p \leq 0.001$; NS, not significant (unpaired Student's t-test). Data are averages of three or more independent experiments.

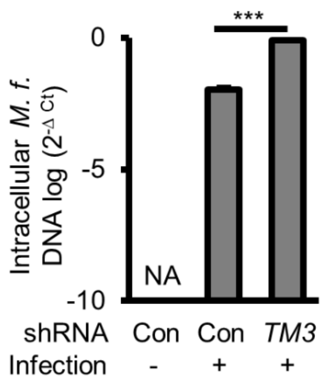

Supplementary Figure S5, Knockdown of h*IFITM3* enhances intracellular levels of *M. fermentans* in SH-SY5Y cells. Bar graph of intracellular *M. fermentans* DNA levels normalized against an internal control in *M. fermentans*-infected SH-SY5Y cells with indicated gene knockdown at 12 dpi, determined by qPCR. ***$p \leq 0.001$ (unpaired Student's t-test); NA, not applicable; Con, control knockdown SH-SY5Y cells; *TM3*, h*IFITM3* knockdown SH-SY5Y cells. Data are averages of three or more independent experiments.

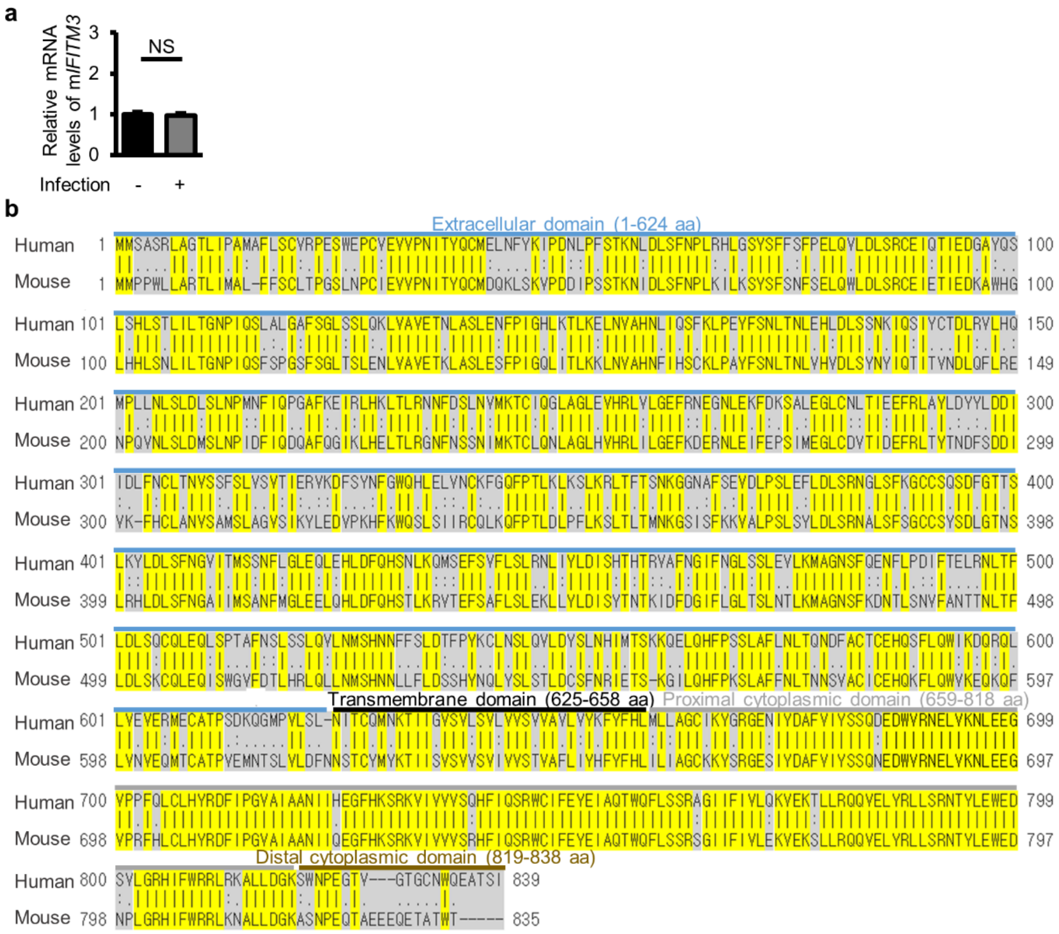

Supplementary Figure S6. *M. fermentans* does not upregulate mouse *IFITM3* gene expression levels via TLR4. (**a**) Bar graph of mouse *IFITM3* gene expression levels normalized against the *GAPDH* gene in mock- or *M. fermentans*-infected neuro2a cells at 12 dpi. (**b**) Amino acid alignment of human and mouse TLR4 homologs. Amino acid sequences for human TLR4 (NCBI Reference Sequence: NP_612564.1) and mouse TLR4 (NCBI Reference Sequence: NP_067272.1) were aligned with pairwise sequence alignment tools based on the EMBOSS Needle using the Needleman-Wunsch algorithm. Yellow indicates 100% identity at a given position, and gray is used elsewhere. Bar graphs present mean values ± SD; NS, not significant (unpaired Student's t-test). Data are averaged from more than three independent experiments.

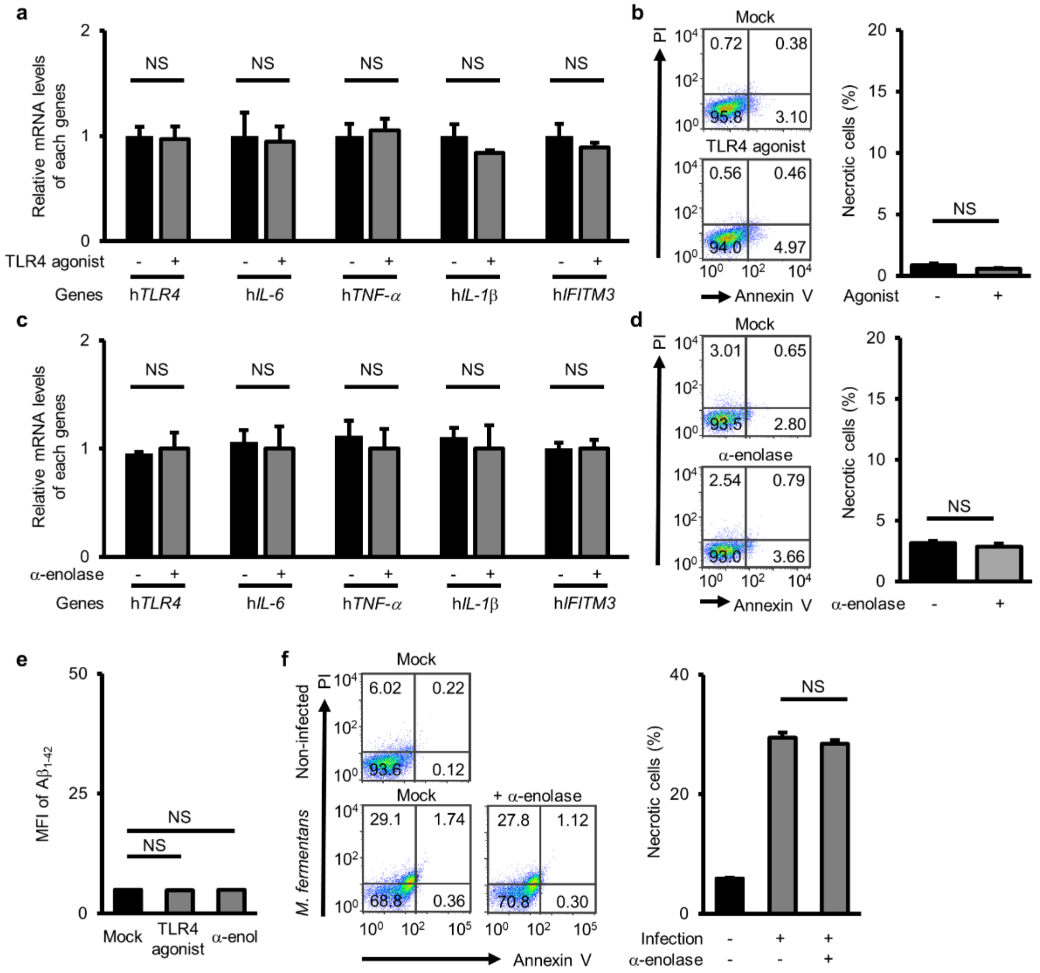

Supplementary Figure S7. Absence of TLR4 signaling activation by CRX-527 or α-enolase in SH-SY5Y Cells. (**a**) Bar graph of indicated gene expression levels normalized against *GAPDH* gene in SH-SY5Y cells treated with 1 μg/ml of TLR4 agonist (CRX-527) or mock for 24 h, determined by qPCR. (**b**) Representative flow cytometry dot plots from Annexin V/PI apoptosis assays for cells from (**a**), and a bar graph of Annexin V-negative PI-positive cells. (**c**) Bar graph of indicated gene expression levels normalized against *GAPDH* gene in SH-SY5Y cells treated with 10 μg/ml of α-enolase or mock for 24 h, determined by qPCR. (**d**) Representative flow cytometry dot plots from Annexin V/PI apoptosis assays for cells from (**c**), and a bar graph of Annexin V-negative PI-positive cells. (**e**) Bar graph for flow cytometry analysis of intracellular $A\beta_{1\text{-}42}$ in SH-SY5Y cells treated with 1 μg/ml of TLR4 agonist or 10 μg/ml of α-enolase for 24 h. (**f**) Representative flow cytometry dot plots from Annexin V/PI apoptosis assays of non- or *M. fermentans*-infected SH-SY5Y cells after treatment with 10 μg/ml of α-enolase or mock. Bar graphs present mean values ± SD; NS, not significant (unpaired Student's t-test); α-enol, α-enolase; MFI, mean fluorescence intensity. Data are averaged from more than three independent experiments.

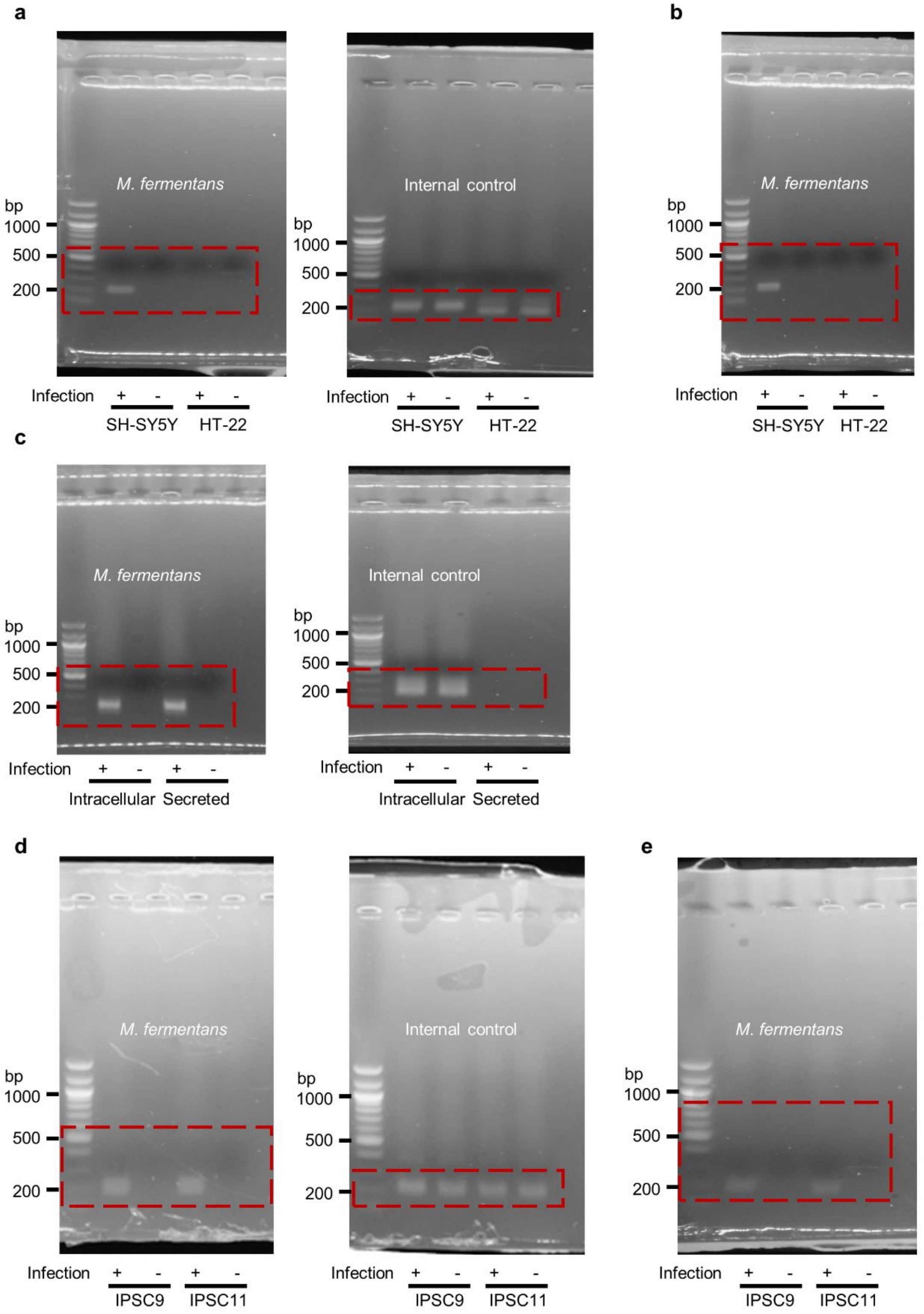

Supplementary Figure S8. Original full-length gel images. (**a-e**) Original gel images of (**a**) Supplementary Fig. S1a; (**b**) Supplementary Fig. S1b; (**c**) Supplementary Fig. S1c; (**d**) Supplementary Fig. S3b; (**e**) Supplementary Fig. S3d.

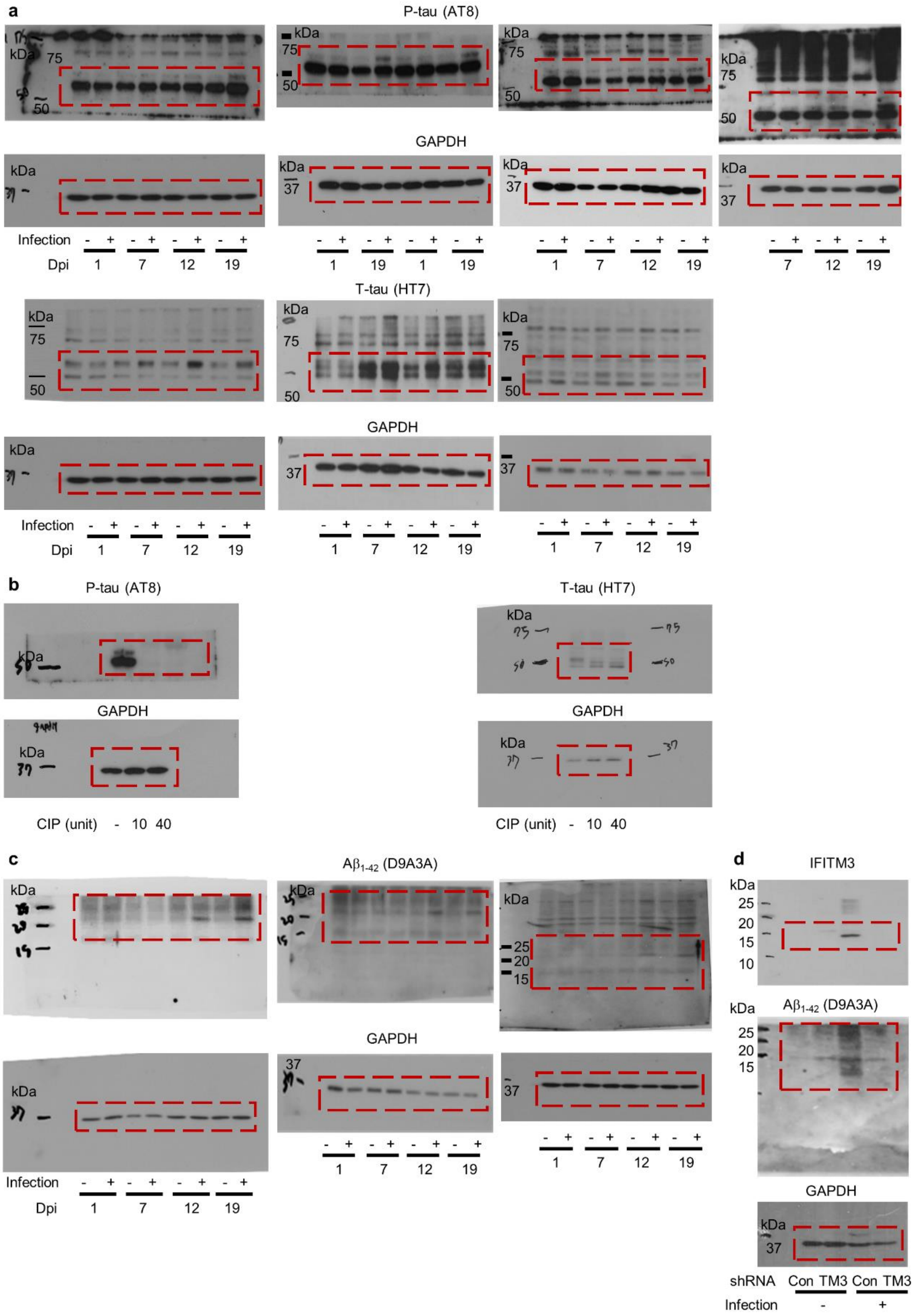

Supplementary Figure S9. Original blots of western blot analysis. (**a-d**) Original blot image of (**a**) Figure 2a; (**b**) Figure 2d; (**c**) Figure 2e; (**d**) Figure 5d. It is important to note

that the original blots were cut before hybridization with antibodies. For this reason, full-length blots cannot be provided. The images depict the blots after cropping.

Supplementary Table S1. Odds ratio of *M. fermentans* infection in blood samples of neurological disease patients.

Disease	Patients (%)	Control (%)	Odds ratio (95% CI)	*p*-value	Method	Reference
CFS	32/100 (32)	8/100 (8)	5.4 (2.3–12.5)	<0.001	PCR of PBMC	1
CFS	67/261 (26)	1/36 (3)	12.1 (1.6–90)	0.015	PCR of blood	2
CFS	44/91 (48)	0/32 (0)	60.9 (3.6–1024.6)	0.004	PCR of blood	3
GWV family with CFS	36/57 (63)	2/50 (4)	41.1 (9.1–186.9)	<0.001	PCR of blood	4
CFS	32/100 (32)	13/160 (8)	5.3 (2.6–10.8)	<0.001	PCR of PBMC	5
GWS	22/60 (36)	13/160 (8)	6.5 (3–14.2)	<0.001	PCR of PBMC	5
GWS	38/110 (35)	2/70 (3)	17.9 (4.2–77.3)	<0.001	PCR of blood	4
Civilians with ALS	13/28 (46)	2/70 (3)	29.5 (6–144.5)	<0.001	PCR of blood	6
GWV with ALS	7/8 (88)	2/70 (3)	238 (19.1–2968.2)	<0.001	PCR of blood	6
ALS	6/13 (46)	4/44 (9)	8.6 (1.9–38.4)	0.005	PCR of blood	7
ASD	17/48 (35)	0/45 (0)	50.6 (2.9–871.9)	0.007	PCR of blood	8

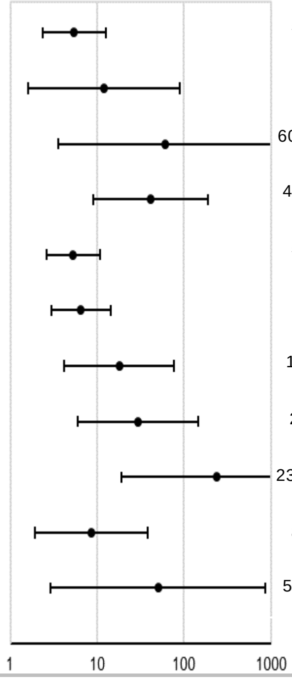

CFS, Chronic fatigue syndrome; FMS, Fibromyalgia; GWS, Gulf War syndrome; ALS, Amyotrophic lateral sclerosis; ASD, Autism Spectrum Disorder; GWV, Gulf War veterans.

Supplementary references

1 Choppa, P. C., Vojdani, A., Tagle, C., Andrin, R. & Magtoto, L. Multiplex PCR for the detection of Mycoplasma fermentans, M. hominis and M. penetrans in cell cultures and blood samples of patients with chronic fatigue syndrome. *Mol. Cell. Probes* **12**, 301-308, doi:10.1006/mcpr.1998.0186 (1998).

2 Nijs, J., Nicolson, G. L., De Becker, P., Coomans, D. & De Meirleir, K. High prevalence of Mycoplasma infections among European chronic fatigue syndrome patients. Examination of four Mycoplasma species in blood of chronic fatigue syndrome patients. *FEMS Immunol. Med. Microbiol.* **34**, 209-214, doi:10.1111/j.1574-695X.2002.tb00626.x (2002).

3 Nasralla, M., Haier, J. & Nicolson, G. L. Multiple mycoplasmal infections detected in blood of patients with chronic fatigue syndrome and/or fibromyalgia syndrome. *Eur. J. Clin. Microbiol. Infect. Dis.* **18**, 859-865, doi:10.1007/s100960050420 (1999).

4 Garth L. Nicolson, M. Y. N., Nancy L. Nicolson & Joerg Haier. High Prevalence of Mycoplasma Infections in Symptomatic (Chronic Fatigue Syndrome) Family Members of Mycoplasma-Positive Gulf War Illness Patients. *Journal of Chronic Fatigue Syndrome* **11**, 21-36, doi:10.1300/J092v11n02_03 (2003).

5 Vojdani, A. & Franco, A. R. Multiplex PCR for the Detection of Mycoplasma fermentans, M. hominis, and M. penetrans in Patients with Chronic Fatigue Syndrome, Fibromyalgia, Rheumatoid Arthritis, and Gulf War Syndrome. *Journal of Chronic Fatigue Syndrome* **5**, 187-197, doi:10.1300/J092v05n03_16 (1999).

6 Nicolson, G. L., Nasralla, M. Y., Haier, J. & Pomfret, J. High frequency of systemic mycoplasmal infections in Gulf War veterans and civilians with Amyotrophic Lateral Sclerosis (ALS). *J. Clin. Neurosci.* **9**, 525-529, doi:10.1054/jocn.2001.1075 (2002).

7 Gil, C. *et al.* Detection of Mycoplasmas in Patients with Amyotrophic Lateral Sclerosis. *Advances in Microbiology*, 712-719 (2014).

8 Nicolson, G. L., Gan, R., Nicolson, N. L. & Haier, J. Evidence for Mycoplasma ssp., Chlamydia pneunomiae, and human herpes virus-6 coinfections in the blood of patients with autistic spectrum disorders. *J. Neurosci. Res.* **85**, 1143-1148, doi:10.1002/jnr.21203 (2007).
